# Supplementary material for: Predictors to Intensive Care Unit admission among patient with coronavirus disease in Sukraraj Tropical and Infectious Disease Hospital, Nepal: A case-control study
Source: PLOS Glob Public Health. 2024 Mar 21;4(3):e0002516. doi: 10.1371/journal.pgph.0002516 (PMC10957074; doi:10.1371/journal.pgph.0002516)
Supplement: S1 Text — (DOCX) [file pgph.0002516.s001.docx]

| SN | Item | Options | | | | |
| --- | --- | --- | --- | --- | --- | --- |
| 1 | How often do you shake hands while greeting people nowadays? | Always (more than 90% times) | Mostly (Approx 75% times) | Commonly (Approx 50% times) | Occasionally  (approx. 25% times) | Rarely (less than 10% times) |
| 2 | How often do you wash/sanitize your hands with soap and water/alcohol-based sanitizer? | Always (more than 90% times) | Mostly (Approx 75% times) | Commonly (Approx 50% times) | Occasionally  (approx. 25% times) | Rarely (less than 10% times) |
| 3 | How often do you ensure that you wash/sanitize your hands for at least 20s? | Always (more than 90% times) | Mostly (Approx 75% times) | Commonly (Approx 50% times) | Occasionally  (approx. 25% times) | Rarely (less than 10% times) |
| 4 | How often do you ensure that you cover your face with a handkerchief/bent elbow while coughing/sneezing? | Always (more than 90% times) | Mostly (Approx 75% times) | Commonly (Approx 50% times) | Occasionally  (approx. 25% times) | Rarely (less than 10% times) |
| 5 | How often do you ensure that you clean your hands before touching your eyes/nose/mouth? | Always (more than 90% times) | Mostly (Approx 75% times) | Commonly (Approx 50% times) | Occasionally  (approx. 25% times) | Rarely (less than 10% times) |
| 6 | How often do you maintain a minimum distance of one meter at your workplace? | Always (more than 90% times) | Mostly (Approx 75% times) | Commonly (Approx 50% times) | Occasionally  (approx. 25% times) | Rarely (less than 10% times) |
| 7 | How often do you maintain a minimum distance of one meter while eating food with your colleagues at your workplace? | Always (more than 90% times) | Mostly (Approx 75% times) | Commonly (Approx 50% times) | Occasionally  (approx. 25% times) | Rarely (less than 10% times) |
| 8 | How often do you avoid going out of the house unnecessarily? | Always (more than 90% times) | Mostly (Approx 75% times) | Commonly (Approx 50% times) | Occasionally  (approx. 25% times) | Rarely (less than 10% times) |
| 9 | How often have you attended social gathering in the past two months? (Like meeting friends, going to religious places, visiting malls, theaters etc) | Never (5) | Once (4) | Twice (3) | Thrice (2) | More than three times (1) |
| 10 | How often do you maintain distance of one meter in public spaces (eg grocery shopping, social gathering etc) ? | Always (more than 90% times) | Mostly (Approx 75% times) | Commonly (Approx 50% times) | Occasionally  (approx. 25% times) | Rarely (less than 10% times) |
| 11 | How often do you wear masks while going out of home? | Always (more than 90% times) | Mostly (Approx 75% times) | Commonly (Approx 50% times) | Occasionally  (approx. 25% times) | Rarely (less than 10% times) |
| 12 | While wearing a mask, how often do you ensure that both your nose and mouth are covered? | Always (more than 90% times) | Mostly (Approx 75% times) | Commonly (Approx 50% times) | Occasionally  (approx. 25% times) | Rarely (less than 10% times) |
| 13 | How often do you keep your mask properly in a separate bag/dustbin after using it? | Always (more than 90% times) | Mostly (Approx 75% times) | Commonly (Approx 50% times) | Occasionally  (approx. 25% times) | Rarely (less than 10% times) |
| 14 | How often do you sanitize your personal items (eg purse/mobile phone, etc) with sanitizer when you come home? | Always (more than 90% times) | Mostly (Approx 75% times) | Commonly (Approx 50% times) | Occasionally  (approx. 25% times) | Rarely (less than 10% times) |
| 15 | How often do you take precautions when buying things to avoid virus contamination? | Always (more than 90% times) | Mostly (Approx 75% times) | Commonly (Approx 50% times) | Occasionally  (approx. 25% times) | Rarely (less than 10% times) |
| 16 | How often do you obey government restrictions regarding the COVID-19 pandemic? | Always (more than 90% times) | Mostly (Approx 75% times) | Commonly (Approx 50% times) | Occasionally  (approx. 25% times) | Rarely (less than 10% times) |
| 17 | In case you develop symptoms of the disease, you will contact the hospital/helpline/authority regarding it? | Strongly agree | Agree | Can’t say | Disagree | Strongly disagree |
| 18 | If you come in contact with COVID positive/suspect person, you would stop going to work and confine yourself to the home away from friends and family members. | Strongly agree | Agree | Can’t say | Disagree | Strongly disagree |
