# Supplementary material for: Predictors to Intensive Care Unit admission among patient with coronavirus disease in Sukraraj Tropical and Infectious Disease Hospital, Nepal: A case-control study
Source: PLOS Glob Public Health. 2024 Mar 21;4(3):e0002516. doi: 10.1371/journal.pgph.0002516 (PMC10957074; doi:10.1371/journal.pgph.0002516)
Supplement: S2 Text — (DOCX) [file pgph.0002516.s002.docx]

**Nepali version of Preventive practice questionnaire**

1. आजकल तपाई मानिसहरूलाई अभिवादन गर्दा कति पटक हात मिलाउनुहुन्छ?

- सधैं (९०% भन्दा बढी पटक)
- प्रायः (लगभग ७५% पटक)
- सामान्यतया (लगभग ५०% पटक)
- कहिलेकाहीँ (लगभग २५% पटक)
- विरलै (१०% पटक भन्दा कम)

1. तपाइँ कति पटक साबुन र पानी/अल्कोहलमा आधारित सेनिटाइजरले तपाइँको हात धुनुहुन्छ / सेनिटाइज गर्नुहुन्छ ?

- सधैं (९०% भन्दा बढी पटक)
- प्रायः (लगभग ७५% पटक)
- सामान्यतया (लगभग ५०% पटक)
- कहिलेकाहीँ (लगभग २५% पटक)
- विरलै (१०% पटक भन्दा कम)

1. तपाईंले कम्तिमा 20 सेकेन्डसम्म आफ्नो हात धुने/सनिटाइज गर्ने कुरा कति पटक सुनिश्चित गर्नुहुन्छ?

- सधैं (९०% भन्दा बढी पटक)
- प्रायः (लगभग ७५% पटक)
- सामान्यतया (लगभग ५०% पटक)
- कहिलेकाहीँ (लगभग २५% पटक)
- विरलै (१०% पटक भन्दा कम)

1. तपाईंले खोक्दा/हाछ्युँ गर्दा कति पटक यो सुनिश्चित गर्नुहुन्छ कि तपाईंले आफ्नो अनुहार रुमाल/ कुहिनोले छोप्नु हुन्छ ?

- सधैं (९०% भन्दा बढी पटक)
- प्रायः (लगभग ७५% पटक)
- सामान्यतया (लगभग ५०% पटक)
- कहिलेकाहीँ (लगभग २५% पटक)
- विरलै (१०% पटक भन्दा कम)

1. तपाइँ कति पटक तपाइँको आँखा / नाक / मुख छुनु अघि तपाइँको हातहरू सफा छ/छैन भनेर सुनिश्चित गर्नुहुन्छ ?

- सधैं (९०% भन्दा बढी पटक)
- प्रायः (लगभग ७५% पटक)
- सामान्यतया (लगभग ५०% पटक)
- कहिलेकाहीँ (लगभग २५% पटक)
- विरलै (१०% पटक भन्दा कम)

1. तपाईं आफ्नो कार्यस्थलमा न्यूनतम एक मिटरको दुरी कति पटक कायम गर्नुहुन्छ?

- सधैं (९०% भन्दा बढी पटक)
- प्रायः (लगभग ७५% पटक)
- सामान्यतया (लगभग ५०% पटक)
- कहिलेकाहीँ (लगभग २५% पटक)
- विरलै (१०% पटक भन्दा कम)

1. तपाईको कार्यस्थलमा आफ्ना सहकर्मीहरूसंग खाना खाँदा कति पटक न्यूनतम एक मिटरको दुरी कायम राख्नुहुन्छ ?

- सधैं (९०% भन्दा बढी पटक)
- प्रायः (लगभग ७५% पटक)
- सामान्यतया (लगभग ५०% पटक)
- कहिलेकाहीँ (लगभग २५% पटक)
- विरलै (१०% पटक भन्दा कम)

1. अनावश्यक रूपमा घरबाहिर ननिस्कन कत्तिको बेवास्ता गर्नुहुन्छ ?

- सधैं (९०% भन्दा बढी पटक)
- प्रायः (लगभग ७५% पटक)
- सामान्यतया (लगभग ५०% पटक)
- कहिलेकाहीँ (लगभग २५% पटक)
- विरलै (१०% पटक भन्दा कम)

1. विगत दुई महिनामा तपाईले कति पटक सामाजिक जमघटमा भाग लिनुभयो? (जस्तै बैठक,

साथीहरू भेट्ने , धार्मिक स्थलहरूमा जाने, मल, थिएटरहरू, आदि)

- कहिले पनि छैन
- एक चोटी
- दुई चोटी
- तिन चोटी
- तिन भन्दा धेरै

1. तपाईले कति पटक सार्वजनिक स्थानहरूमा न्यूनतम एक मिटरको दूरी कायम गर्नुहुन्छ (जस्तै।

किराना किनमेल, सामाजिक जमघट, आदि)?

- सधैं (९०% भन्दा बढी पटक)
- प्रायः (लगभग ७५% पटक)
- सामान्यतया (लगभग ५०% पटक)
- कहिलेकाहीँ (लगभग २५% पटक)
- विरलै (१०% पटक भन्दा कम)

1. घरबाहिर निस्कँदा कति पटक मास्क लगाउनुहुन्छ ?

- सधैं (९०% भन्दा बढी पटक)
- प्रायः (लगभग ७५% पटक)
- सामान्यतया (लगभग ५०% पटक)
- कहिलेकाहीँ (लगभग २५% पटक)
- विरलै (१०% पटक भन्दा कम)

1. मास्क लगाउँदा, तपाइँ कति पटक यो सुनिश्चित गर्नुहुन्छ कि तपाइँको नाक र मुख दुबै छ

ढाकिएको छ ?

- सधैं (९०% भन्दा बढी पटक)
- प्रायः (लगभग ७५% पटक)
- सामान्यतया (लगभग ५०% पटक)
- कहिलेकाहीँ (लगभग २५% पटक)
- विरलै (१०% पटक भन्दा कम)

1. तपाईंले कति पटक आफ्नो मास्क प्रयोग गरिसकेपछि अलग्गै झोला/डस्टबिनमा राम्ररी राख्नुहुन्छ?

- सधैं (९० % भन्दा बढी पटक)
- प्रायः (लगभग ७५% पटक)
- सामान्यतया (लगभग ५०% पटक)
- कहिलेकाहीँ (लगभग २५% पटक)
- विरलै (१०% पटक भन्दा कम)

1. तपाई घर आइसके पछि आफ्नो व्यक्तिगत वस्तुहरू (जस्तै पर्स/मोबाइल फोन, इत्यादि) कति पटक सेनिटाइजरले सेनिटाइज गर्नुहुन्छ ?

- सधैं (९०% भन्दा बढी पटक)
- प्रायः (लगभग ७५% पटक)
- सामान्यतया (लगभग ५०% पटक)
- कहिलेकाहीँ (लगभग २५% पटक)
- विरलै (१०% पटक भन्दा कम)

1. सामान किन्दा भाइरस संक्रमणबाट बच्न कति पटक सावधानी अपनाउनुहुन्छ?

- सधैं (९०% भन्दा बढी पटक)
- प्रायः (लगभग ७५% पटक)
- सामान्यतया (लगभग ५०% पटक)
- कहिलेकाहीँ (लगभग २५% पटक)
- विरलै (१०% पटक भन्दा कम)

1. कोभिड-१९ महामारीको सन्दर्भमा सरकारी प्रतिबन्धहरूको कत्तिको पालना गर्नुहुन्छ?

- सधैं (९०% भन्दा बढी पटक)
- प्रायः (लगभग ७५% पटक)
- सामान्यतया (लगभग ५०% पटक)
- कहिलेकाहीँ (लगभग २५% पटक)
- विरलै (१०% पटक भन्दा कम)

1. यदि तपाईंलाई रोगको लक्षणहरू देखा परेमा, तपाईंले यस सम्बन्धमा अस्पताल/हेल्पलाइन/ अख्तियार मा सम्पर्क गर्नुहुनेछ ?

- एकदमै सहमत
- सहमत
- भन्न सक्दिन
- असहमत
- एकदमै असहमत

1. दि तपाइँ कोभिड पोजिटिभ/शङ्कास्पद व्यक्तिको सम्पर्कमा आउनुभयो भने, तपाइँ काममा जान बन्द गरि आफूलाई साथीहरू र परिवारका सदस्यहरूबाट टाढा घरमा सीमित राख्नु हुन्छ ?

- एकदमै सहमत
- सहमत
- भन्न सक्दिन
- असहमत
- एकदमै असहमत

• अन्य कारणहरू (कृपया निर्दिष्ट गर्नुहोस्):
