# Supplementary material for: Predictors to Intensive Care Unit admission among patient with coronavirus disease in Sukraraj Tropical and Infectious Disease Hospital, Nepal: A case-control study
Source: PLOS Glob Public Health. 2024 Mar 21;4(3):e0002516. doi: 10.1371/journal.pgph.0002516 (PMC10957074; doi:10.1371/journal.pgph.0002516)
Supplement: Attachment — Submitted filename: PGPH-D-23-00924_reviewer_2.pdf [file pgph.0002516.s005.pdf]

PLOS Global Public Health

PREDICTORS TO INTENSIVE CARE UNIT ADMISSION AMONG PATIENT WITH CORONAVIRUS DISEASE IN SUKRARAJ TROPICAL AND INFECTIOUS DISEASE HOSPITAL, NEPAL: A CASE CONTROL STUDY

--Manuscript Draft--

**Manuscript Number:**	PGPH-D-23-00924R1
**Article Type:**	Research Article
**Full Title:**	PREDICTORS TO INTENSIVE CARE UNIT ADMISSION AMONG PATIENT WITH CORONAVIRUS DISEASE IN SUKRARAJ TROPICAL AND INFECTIOUS DISEASE HOSPITAL, NEPAL: A CASE CONTROL STUDY
**Short Title:**	Predictors to Intensive care unit admission
**Corresponding Author:**	Dipsikha Aryal, Masters in Public Health<br>BP Koirala Institute of Health Sciences<br>Dharan, Province 1, Nepal NEPAL
**Order of Authors:**	Dipsikha Aryal, Masters in Public Health
	Paras Kumar Pokharel, MD in Community Medicine
	Anup Ghimire, MD in Community Medicine
	Vijay Kumar Khanal, MD in Community Medicine
	Gyanu Nepal Gurung, Masters in Public Health
	Bimal Sharma Chalise, MD
	Sudikshya Neupane, Masters in Public Health
	Shikha Basnet
**Keywords:**	Keywords: COVID-19, Intensive care unit, Preventive practices
**Abstract:**	Introduction<br>The clinical features of COVID-19 are varied, ranging from asymptomatic states or mild upper respiratory tract infections to severe pneumonia with respiratory failure acute respiratory distress syndrome in the early stages, or even death. Studies have shown that 20.0% of patients require hospitalization with 10.0–20.0% being admitted to the intensive care unit. This study aimed to assess predictors associated with COVID-19 leading to ICU admission among reverse transcriptase- polymerase chain reaction positive patients in Sukraraj Tropical and infectious disease hospital, Nepal.<br>Methods<br>We conducted a case-control study from June 2022 to July 2022 among patients admitted to Sukraraj Tropical and Infectious Disease Hospital. Hospital-based age (± 2 years) and sex-matched case-control study design was adopted in which ICU admitted (case group, n= 33) and General ward admitted (control group, n=66) were taken. We collected data through a review of hospital files and telephone interviews using a structured questionnaire consisting of socio-demographics, clinical and preventive predictors. Data were analyzed using the statistical package for social science version 11.5. We present parametric numerical variables as mean and standard deviation, and categorical variables as percentage and frequency. We applied the Chi-square test and conditional logistics regression to determine the predictors associated with ICU admission.<br>Results<br>High blood pressure, high C-reactive protein and poor application of preventive practice were found to be the predictors of ICU admission. The conditional logistics regression analyses revealed that independent risk factors associated with ICU admission were elevated blood pressure (AOR = 2.22; 95% CI 1.05 – 4.71, p 2.2= 0.015), abnormal CRP (AOR = 2.92; 95% CI 1.24 – 6.84, p = 0.012) at the time of hospital admission were more likely to get admitted to ICU. Patient with poor preventive practice (AOR = 3.34; 95% CI 1.19 – 9.31, p=0.02) more likely to get admitted to ICU than patient with good preventive practice.

Powered by Editorial Manager® and ProduXion Manager® from Aries Systems Corporation

	Conclusion: These findings could be utilized for early triage and risk assessment in COVID-19 patients. Developing evidence-based and appropriate community-based preventive practice awareness programs among the general public is recommended.<br>Keywords: COVID-19, Intensive care unit, Preventive practices
**Additional Information:**	
**Question**	**Response**
**Financial Disclosure**<br><br>Enter a financial disclosure statement that describes the sources of funding for the work included in this submission and the role the funder(s) played. This includes grants and any commercial funding of the work or authors.<br><br>This statement will be typeset if the manuscript is accepted for publication.<br><br>*Please review the submission guidelines and the instructions link below for detailed requirements and guidance.*	The authors received no specific funding for this work.
**Competing Interests**<br><br>On behalf of all authors, disclose any competing interests that could be perceived to bias this work.<br><br>This statement will be typeset if the manuscript is accepted for publication.<br><br>*Please review the instructions link below and PLOS Global Public Health's competing interests policy to determine what information must be disclosed at submission.*	The authors have declared that no competing interests exist.
**Data Availability**<br><br>Before publication, Authors are required to make fully available and without restriction all data underlying their findings. Please see our PLOS Data Policy page for detailed information on	Data can be found in .osf, link of data osf attached below: https://osf.io/az4wr/

this policy.<br><br>A **Data Availability Statement**, detailing where the data can be accessed, is required at first submission. Insert your Data Availability Statement in the box below.<br><br>Please see the data reporting section of our submission guidelines for instructions on what you need to include in your Data Availability Statement.<br><br>This statement will be typeset if the manuscript is accepted for publication.<br><br>*PLOS allows rare exemptions to address legal and ethical concerns. If you have legal or ethical restrictions, please use the box below to detail these in full sentences for the Journal team to consider.*	

Manuscript

Click here to access/download;Manuscript;original manuscript.docx

PREDICTORS TO INTENSIVE CARE UNIT ADMISSION AMONG PATIENT WITH CORONAVIRUS DISEASE IN SUKRARAJ TROPICAL AND INFECTIOUS DISEASE HOSPITAL, NEPAL: A CASE CONTROL STUDY

Dipsikha Aryal[1], Paras Kumar Pokharel[1], Anup Ghimire[1], Vijay Kumar Khanal[1], Gyanu Nepal Gurung[1], Bimal Sharma Chalise[2], Sudikshya Neupane[1], Shikha Basnet[1]

Affiliations

[1] B.P Koirala Institute of Health Sciences, School of Public Health and Community Medicine, Dharan, Nepal

[2] Sukraraj Tropical and Infectious Disease Hospital, Teku, Nepal

Corresponding author: *Dipsikha Aryal*

Phone number: +977- 9840143527

Email ID: seekuary@gmail.com

ABSTRACT

Introduction

The clinical features of COVID-19 are varied, ranging from asymptomatic states or mild upper respiratory tract infections to severe pneumonia with respiratory failure acute respiratory distress syndrome in the early stages, or even death. Studies have shown that 20.0% of patients require hospitalization with 10.0–20.0% being admitted to the intensive care unit. This study aimed to assess predictors associated with COVID-19 leading to ICU admission among reverse transcriptase- polymerase chain reaction positive patients in Sukraraj Tropical and infectious disease hospital, Nepal.

Methods

We conducted a case-control study from June 2022 to July 2022 among patients admitted to Sukraraj Tropical and Infectious Disease Hospital. Hospital-based age (± 2 years) and sex-matched case-control study design was adopted in which ICU admitted (case group, n= 33) and General ward admitted (control group, n=66) were taken. We collected data through a review of hospital files and telephone interviews using a structured questionnaire consisting of socio-demographics, clinical and preventive predictors. Data were analyzed using the statistical package for social science version 11.5. We present parametric numerical variables as mean and standard deviation, and categorical variables as percentage and frequency. We applied the Chi-square test and binary logistics regression to determine the predictors associated with ICU admission.

Results

High blood pressure, high C-reactive protein, poor application of preventive practice and longer duration of hospital stay were found to be the predictors of ICU admission. The adjusted

multivariable logistics regression analyses revealed that independent risk factors associated with ICU admission were high blood pressure at the time of hospital admission (AOR = 9.88; 95% CI: 1.4 – 68.90, p= 0.021), high C reactive protein (AOR = 12.23; 95% CI: 1.37 – 108.53, p= 0.025), poor preventive practice (AOR= 16.22; 95% CI: 2.26 – 116.40, p= 0.006), duration of hospital stay (AOR = 1.45; 95% CI: 1.17 – 1.79, p= 0.001).

Conclusion: These findings could be utilized for early triage and risk assessment in COVID-19 patients. Developing evidence-based and appropriate community-based preventive practice awareness programs among the general public is recommended.

Keywords: COVID-19, Intensive care unit, Preventive practices

INTRODUCTION

Coronavirus disease 2019 (COVID-19) caused by the severe acute respiratory syndrome coronavirus 2 (SARS-CoV-2) previously known as 2019-nCoV which was first identified in Wuhan China in December 2019 and World Health Organization declared a pandemic in March 2020(1).

The clinical features of COVID-19 are varied, ranging from asymptomatic states or mild upper respiratory tract infections to severe pneumonia with respiratory failure acute respiratory distress syndrome in the early stages or even death. Studies have shown that major symptoms like fever, cough, dyspnea and minor symptoms such as loss of taste and smell, gastrointestinal symptoms and cutaneous manifestations are common clinical features of this disease (2).

The majority of patients progress favorably. However, 20% of patients require hospitalization and their disease can progress rapidly to acute respiratory distress syndrome (ARDS), multiple organ dysfunction syndrome, or even death (3). Rates for the cases requiring intensive care are low, with 10–20% being admitted to the intensive care unit (ICU), 3–10% requiring intubation, and 2–5% dead. Severe patients show respiratory failure, pneumonia, multi-organ failure, and shock, which require care in intensive care facilities (4).

The first case of coronavirus was seen in a Nepalese Citizen returning from Wuhan City returned on 9 January 2020 and visited Sukraraj Tropical and Infectious Disease Hospital on 13 January 2020. National Public Health Laboratory (NPHL) communicated the positive test result for 2019-nCoV on 23 January 2020 (5). On 23rd March 2020, a second case of COVID-19 was seen in Nepal. Immediately after the confirmation of the second case nationwide lockdown was endorsed in Nepal from 25th March 2020 (6). Studies show the basic reproductive number during the second

wave was larger than the first wave (7). The expected cases from September 2021 to April 2022 were 111,300 among which 3590 need ICU (7).

A variety of containment and mitigation strategies have been adopted in several countries to adequately respond to covid-19, with the intention of deferring major surges of patients in hospitals and protecting the most vulnerable people from infection, COVID-19 clinical management guidelines, guidelines for the use of personal protective equipment, guidelines for waste management including elderly people and those with comorbidities (8). Several factors like diverse cultures, wide socioeconomic disparities and healthcare services inequalities might pose challenges in the implementation of these preventive practices at the community level (9).

In Nepal, to date, most clinical studies on COVID-19 have focused on describing the general epidemiologic and clinical characteristics. Studies are mainly focused on comparing the association between survivor and non – survivor group, RT PCR for COVID-19 test positive and negative group but data on comparison between ICU admitted and non-admitted group is still lacking. However, combined data on how the risks associated with demographic, underlying co-morbidities, clinical and public health predictors in this setting is limited. In this context, we conducted a study to assess predictors associated with COVID-19 leading to ICU admission among RT PCR positive for SARS-COV-2 patients in Sukraraj Tropical and Infectious Disease Hospital.

MATERIALS AND METHODS

Study Design, population and setting

We conducted a hospital-based (age +/- 2 years and sex) matched case-control study, where proposal writing started from 1st April 2022, followed by data collection from 24th June 2022 to 12th July 2022 among Reverse transcriptase polymerase chain reaction positive for SARS-COV-2 patients admitted to covid ICU and general ward of Sukraraj Tropical and Infectious Disease Hospital.

Sample size

We calculated the sample size for this study by taking reference to case-control study done in Spain on 2021 (3), the proportion of cases (p_1) is 33.33% and the proportion of control (p_2) was 66.67%. Taking a 95% confidence interval and 80% power of test by using the formula of two proportion the calculated sample size was 33. A sample of 33 cases and 66 control was chosen in (1:2 ratio), the total number of samples was 99.

Sampling technique

Sukraraj Tropical and Infectious Disease Hospital was selected from the list of public hospital with High Dependency Unit, Intensive Care Unit and ventilators in Kathmandu Valley. A list of COVID-19 RT-PCR positive patients admitted to ICU and general ward (Covid ward/ Covid isolation ward) in Sukraraj Tropical and Infectious Disease Hospital from Shrawan to Poush 2078 BS (16th July 2021 – 14th January 2022) was collected. The complete frame of ICU admitted patients which was 222 during six months was divided by the calculated sample size for this study which was 99. Each 2.24 ~ 3rd ICU admitted patient file was reviewed, that baseline characteristics, oxygen saturation, blood pressure, pulse rate, respiration rate and temperature at the time of hospital admission and laboratory electronic record Total Leucocyte Count (TLC), C-Reactive Protein (CRP) and Cycle threshold value (CT value) were recorded. Systematic random sampling

was used for the selection of cases, if the selected 3rd respondents did not meet the inclusion criteria, then the next file was reviewed.

For a selection of control, COVID-19 RT-PCR positive control having at least one or more symptoms during the clinical course of the disease and admitted in general ward was selected according to age and sex-matched with the case. For collecting data on preventive measures, the patient was contacted via phone call.

Among 713 total COVID-19 patients admitted during six months, 2.1% of participants were not eligible for the study because of age limitation. Out of 698, 222 were admitted to ICU and 371 were admitted to COVID-19 general ward. Among 698, 105 (15.04%) were excluded due to death during disease treatment. Out of 593, 143 files were reviewed and data was recorded and each participant was contacted. Among 143 contacted, 2 (1.39%) were excluded due to death after discharge from the hospital. From the remaining 141 participants, 33 cases were matched with 66 control and the remaining 42 unmatched participants were also excluded from this study.

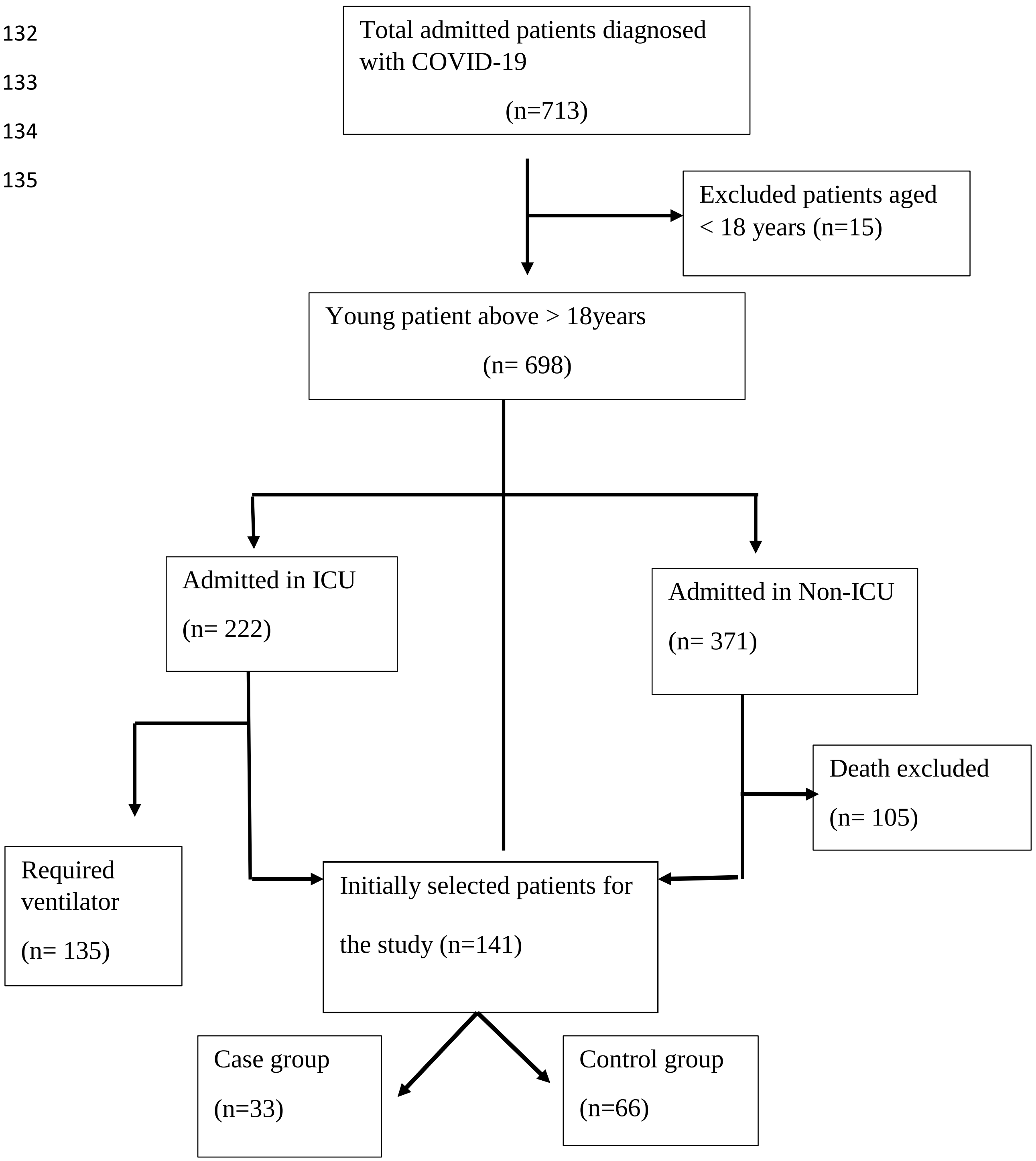

Figure 1 Flowchart of patient enrollment

Ethical approval

We obtained Ethical clearance from the Institutional ethical review committee of B.P. Koirala Institute of Health Sciences (Reference number: 236/078/079-IRC) before conducting the study. Study permission was taken from Sukraraj Tropical and Infectious Disease Hospital (Reference number: 078/79 444) before commencing the study. Due to retrospective nature of study, it was not possible to visit each respondent, therefore verbal consent was taken from the respondents before enrolling them in the study. The confidentiality and anonymity of the participants were maintained and assured throughout the study.

Data collection

Data was collected in a retrospective way using a structured questionnaire that consists of three sections- a) Socio-demographics characteristics; b) Clinical predictors; c) Preventive predictors.

Assessment of socio-demographic and clinical Predictors

Clinical data at hospital admission, including demographic information, chronic comorbidities, vital signs, symptoms, diagnosis of severity of COVID-19, date of admission, date of discharge, COVID-19 vaccination status and outcomes were extracted from medical records using a data collection form.

The severity at admission was categorized into four groups: mild, moderate, severe, and critical, based on the World Health Organization (WHO) COVID-19 Clinical Management Guidelines (Living Guidance 25 January 2021) (10,11).

Lab parameters including threshold cycle CT value, Total Leucocyte Count and C-reactive protein were recorded from an electronic record of a laboratory.

Assessment of preventive predictors

A telephone interview was conducted with each case and control for preventive measures that the patient performed before two weeks of COVID-19 infection. Furthermore, to ascertain the socio-demographics, epidemiological and symptoms data, which were not available from medical records the researcher directly communicated with patients or their families. Other questions include the following components:

Marital status, ethnicity, religion, occupation, family type, family size, means of transportation, smoking and alcohol habit. For preventive practice, a "Questionnaire to assess prevention practice against COVID-19 in general population" was used which was developed by AIIMS New Delhi India.

Description of Preventive practice questionnaire

"Questionnaire to assess prevention practice against COVID-19 in general population" Was developed by AIIMS New Delhi India (12). (Please refer to Annexure – I and Annexure – II for the Questionnaire form in English and Nepali). Preventive practice against COVID-19 pandemic in general population consists of two sections of questions, where the first section assess the preventive practice and the second section assesses the reasons for reasons why preventive practice isn't being followed. Section A consists of 18 questions where question 1 is related to hand, 2, 3 and 5 are related with hand hygiene, question 6, 7 and 10 are related to social distancing, questions 11-13 are related with mask use, questions 4, 14 and 15 are related with basic precautions, questions 8,9, 16 are related with safety and following government restrictions and 17, 18 are

related with health care behavior. For this study, only section A of the questionnaire was used.

Statistical analysis

Data was collected in a paper-based questionnaire which was entered in Statistical Package for Social Sciences (SPSS) 11.5 version. Case-control matching was performed in SPSS. We present parametric numerical variables as mean and standard deviation, and categorical variables as percentage and frequency. The Kolmogorov – Smirnov test and Shapiro – Wilk test was conducted to evaluate whether the data were normally distributed. We applied the Chi-square test for categorical variables and Mann- Whitney U test to compare the association between numerical variables with ICU admission. Binary logistics regression was done to determine the predictors associated with ICU admission. We presented the results of binary logistic regression using adjusted odds ratios (AOR), their 95% confidence intervals and p-values. We conducted all tests at a 95% confidence level and considered p-values less than 0.05 to be statistically significant.

Results

A total of 99 patients were randomly selected from 713 patients who were hospitalized during the study period in participating centers. Table 1 shows the median age of cases was 59 (42 – 70) years, while the median age of control was 59 (43.25 – 70.00) years. Among married, 35.7% were cases and 64.3% of control. Current residence in municipality (38.5% vs 61.5%, p= 0.255), secondary education (37.5% vs 62.5%, p=0.104), unemployed (34.1% vs 65.9%, p=0.138).

Among Brahmin/ Chhetri, 31.0% were case and 69.0% were control. Hindu accounts for 34.1% cases and 65.9% control. Among nuclear family, 40.0% were case and 60.0% control. socio-demographic variables were not found to be significantly associated with ICU admission.

Table 1: Association between sociodemographic characteristics with ICU admission

Variables	ICU admission (Cases) n (%)	General admission (Control) n (%)	p- value
**Age**	59 (42 – 70)	59 (43.25 – 70)	0.970
**Gender**			
Male	22 (33.3)	44 (66.7)	1.000 [a]
Female	11 (33.3)	22 (66.7)	
**Marital Status**			
Married	30 (35.7)	54 (64.3)	0.373 [b]
Others (Unmarried, Widow)	3 (20.0)	12 (80.0)	
**Permanent Address**			
Bagmati Province	22 (33.3)	44 (66.7)	1.000 [a]
Others (Lumbini, Gandaki)	11 (33.3)	22 (66.7)	
**Current residence**			
Municipality	20 (38.5)	32 (61.5)	0.255 [a]
Others (Metropolitan, Sub metropolitan city)	13 (27.7)	34 (72.3)	
**Educational Level**			
Illiterate	3 (15.8)	16 (84.2)	0.104 [b]
Others (Secondary, higher)	30 (37.5)	50 (62.5)	
**Occupation**			
Home maker	8 (47.1)	9 (52.9)	0.138 [c]
Unemployed	15 (34.1)	29 (65.9)	
Employed	10 (26.3)	28 (73.7)	
**Health Professionals**			
Yes	0 (0.0)	4 (100.0)	0.298 [b]
No	33 (34.7)	62 (65.3)	
**Ethnicity**			
Brahmin/Chhetri	18 (31.0)	40 (69.0)	0.564 [a]
Others (Dalit, Newar)	15 (36.6)	26 (63.4)	
**Religion**			
Hindu	31 (34.1%)	60 (65.9)	0.715 [b]
Others (Buddhist, Christian)	2 (25.0)	6 (75.0)	
**Family type**			
Nuclear	22 (40.0)	33 (60.0)	0.137 [a]
Others (Joint, Extended)	11 (25.0)	33 (75.0)	

[a] = Pearson chi square
[b] = Fischer's exact test
[c] = Linear by linear chi square

Table 2 shows the association between co-morbidities and behavioural characteristics with ICU admission. Pre-existing co -morbidities including diabetes, hypertension, cardiac disease, COPD and other medical issues were not significantly associated with ICU admission. In behavioural characteristics, smoking was found to be significantly associated ($p = 0.005$) with ICU admission. Smoker was 3.4 (95% CI: 1.4 – 8.3) times likely admitted in ICU than those who were non-smoker.

Table 2: Association between Co-morbidities and behavioral characteristics with ICU admission

Variables	ICU admission (Cases) n (%)	General admission (Control) n (%)	χ2 test p value	Crude odd's ratio (95% CI)
**Pre-existing Co-morbid illness**				
No	14 (25.9)	40 (74.1)	0.087 [a]	2.08 (0.89 – 4.8)
Yes	19 (42.2)	26 (57.8)		
**Diabetes Mellitus**				
No	28 (32.6)	58 (67.4)	0.674 [a]	0.77 (0.23 – 2.57)
Yes	5 (38.5)	8 (61.5)		
**Hypertension**				
No	22 (31.4)	48 (68.6)	0.532 [a]	
Yes	11 (37.9)	18 (62.1)		0.75 (0.30 – 1.85)
**Cardiac disease**				
No	31 (32.3)	65 (67.7)	0.257 [b]	0.23 (0.02 – 2.7)
Yes	2 (66.7)	1 (33.3)		
**COPD**				
No	26 (30.2)	60 (69.8)	0.092 [a]	0.37 (0.11 – 1.21)
Yes	7 (53.8)	6 (46.2)		
**Other medical issues**				
No	31 (33.7)	61 (66.3)	0.782 [b]	0.78 (0.14 – 4.29)
Yes	2 (28.6)	5 (71.4)		
**Smoking**				
Non- smoker	15 (23.4)	49 (76.6)	**0.005** [a]	3.4 (1.4 – 8.3)
Smoker	18 (51.4)	17 (48.6)		
**Alcohol**				
Non- Alcoholic	17 (29.8)	40 (70.2)	0.388 [a]	1.4 (0.62 – 3.36)
Alcoholic	16 (38.1)	26 (61.9)		

[a] = Pearson chi- square

[b] = Fischer's exact test

Table 3 shows the association of clinical characteristics, lab parameter and COVID-19 vaccination with ICU admission. The odds of ICU admission were 20.0 (95% CI: 5.4 – 72.83; p value: <0.001) times higher in patient with low oxygen saturation than normal oxygen. Patients with elevated blood pressure had 6.10 (95% CI: 2.40 – 15.50; p value: <0.001) odds of ICU admission as compared to normal blood pressure. Patients with pulse rate above 100 bpm had 7.75 (95% CI: 2.23 - 26.87; p value: <0.001) times higher odds of ICU admission as compared to normal pulse rate. Febrile patient had 14.44 (95% CI: 1.65 - 125.75; p value: 0.005) chances of ICU admission in comparison with afebrile patients. Patients with tachypnea had 15.5 (95% CI: 3.4 – 70.10; p value: <0.001) higher odds of ICU admission in comparison with normal respiration rate.

Patients with abnormal Total Leucocyte Count had 10.07 (95% CI: 2.94 – 34.4; p value: <0.001) more chance of ICU admission in contrast with normal TLC. Patients with abnormal CRP had 7.18 (95% CI: 2.77 – 18.65; p value: <0.001) chances of ICU admission in comparison with normal CRP.

Patients without COVID-19 vaccination had 6.78 (95% CI: 2.63 – 17.48; p value: <0.001) higher chance of ICU admission in contrast to those vaccinated either first or second dose with any kind of vaccine.

Table 3: Association between clinical and lab parameter with ICU admission

**Variables**	**ICU admission (Case) (n=33) N %**	**General admission (Control) (n=66) N %**	**χ2 test p value**	**Odds Ratio**	**95% CI (Lower-Upper)**
**Oxygen saturation**					
Normal	3 (6.4)	44 (93.6)	**<0.001***	20.00	5.4 - 72.83
Below	30 (57.7)	22 (42.3)			
**Blood Pressure**					
Normal	14 (20.6)	54 (79.4)	**<0.001***	6.10	2.40 – 15.50
Elevated	19 (61.3)	12 (38.7)			
**Pulse rate**					
Normal	22 (26.2)	62 (73.8)	**<0.001***	7.75	2.23 - 26.87
Above	11 (73.3)	4 (26.7)			
**Temperature**					
Afebrile	27 (29.3)	65 (70.7)	**0.005***	14.44	1.65 - 125.75
Febrile	6 (85.7)	1 (14.3)			
**Respiration rate**					
Normal	2 (5.7)	33 (94.3)	**<0.001***	15.5	3.4 – 70.10
Tachypnea	31 (48.4)	33 (51.6)			
**Total leucocyte count**					
Normal	20 (24.4)	62 (75.6)	**<0.001***	10.07	2.94 – 34.4
Abnormal	13 (76.5)	4 (23.5)			
**C-reactive protein**					
Normal	8 (14.8)	46 (85.2)	**<0.001***	7.18	2.77 – 18.65
Abnormal	25 (55.6)	20 (44.4)			
**COVID-19 vaccination**					
Yes	14 (20.3)	55 (79.7)	**<0.001***	6.78	2.63 – 17.48
No	19 (63.3)	11 (36.7)			

Table 4 shows association between preventive practice and ICU admission. This table explains that patient who performed poor preventive practice had 12.97 times higher odds of getting admitted to ICU in comparison with good preventive practice. Good preventive practice was described as the total score above 54 which was the average score among total.

Table 1: Association between preventive practice and ICU admission

Variables	ICU admission Case (n=33) n %	General admission Control (n=66) n %	p value	Odds Ratio	95% CI (Lower-Upper)
**Preventive practice**					
Good practice	6 (18.2%)	49 (74.2%)	**< 0.001***	12.97	4.57 – 36.79
Poor practice	27 (81.8%)	17 (25.8%)			

Table 5 shows, variables that were significantly associated with ICU admission, with a p-value less than or equal to 0.2 in bivariate analysis were taken for multivariate logistics regression analysis. Variables with expected count less than 5 were not included in multivariate logistics regression analysis.

The adjusted multivariable logistics regression analyses revealed that independent risk factors associated with ICU admission were high blood pressure (AOR = 9.88; 95% CI: 1.4 – 68.90, p= 0.021), high C reactive protein (AOR = 12.23; 95% CI: 1.37 – 108.53, p= 0.025), poor preventive practice (AOR= 16.22; 95% CI: 2.26 – 116.40, p= 0.006), duration of hospital stay (AOR = 1.45; 95% CI: 1.17 – 1.79, p= 0.001).

Table 5: Binary logistics regression model

Variables	Categories	β	p value	Adjusted odds ratio	95% CI for OR	
					Lower	Upper
Family type	Nuclear **	Ref.				
	Joint	-1.536	0.119	0.215	0.031	1.482
Smoking	Non- smoker **	Ref.				
	Smoker	1.835	0.060	6.26	0.924	42.47
Blood pressure	Normal **	Ref.				
	Elevated	2.29	**0.021**	9.88	1.41	68.90
C reactive protein	Normal **	Ref.				
	Abnormal	2.50	**0.025**	12.23	1.37	108.53
Vaccination	Vaccinated **	Ref.				
	Un- vaccinated	0.12	0.896	1.12	0.183	6.95
Preventive practice	Good **	Ref.				
	Poor	2.78	**0.006**	16.22	2.26	116.40
Duration of hospital stay		0.372	**0.001**	1.45	1.17	1.79
Occupation	Employed **	Ref.				
	Home maker	-1.08	0.650	2.48	0.003	36.19
	Unemployed	0.40	0.800	1.44	0.066	34.10
Constant		-8.54	0.001	0.001		

Nagelkerke R square = 0.819 ***: Reference, Cat: categories*

Discussion

ICU admission rate among COVID-19 infected patients was found to be 31.13% in this study which was similar to the previous studies carried in Wuhan where 32% of COVID-19 infected patients were admitted to ICU because they required higher level of oxygen (2). Among the data collected from 20 regions the average ICU admission rate was 21.4%, range 9.4% - 45.9% (13). Another study, highlights that 20% of hospitalized patients with COVID-19 experiencing severe symptoms that required intensive care (14). Furthermore, a meta-analysis reported pooled rate of ICU admission was 32% in COVID -19 patients (15). Slight lower proportion of ICU admission was observed in a study done at South Korea, which reported that in total COVID-19 infected 9.5% of patients required ICU care and 4.6% patients actually admitted to ICU (16).

This study did not find any association between co-morbidities like Diabetes mellitus, hypertension, cardiac disease, COPD with ICU admission. Our findings were consistent with a recent studies done in China and United states, that did not found association between co-morbidities with ICU admission (2,17). A consistent findings was found in another study conducted in Spain, in which COPD and Asthma were not associated with ICU admission (3). Another cross- sectional analytical study conducted in tertiary care center of Nepal also found that co-morbidities were not associated with outcome of COVID-19 (18). In contrast to our findings, a study conducted in Milan, Italy and China found that co-morbidities, most commonly hypertension, cardiovascular disease, diabetes, COPD were related to independent risk factors for death in patient with COVID-19 (19,20). A similar study conducted in rural community hospitals eastern Virginia also revealed that diabetes mellitus and hypertension on admission were predictors of clinical deterioration leading to ICU admission (21). A meta-analysis done by Jianhua Hu and Yanggan Wang also highlighted that prevalence of co-morbidities such as hypertension,

CVD and diabetes mellitus in the severe COVID-19 patients was significantly higher than non-severe patients (22). These pathologies have been associated with greater probability of presenting complications and severe form of disease. The reported inconsistent and conflicting findings might be due to variable COVID-19 severity and mortality based on medical conditions specific to geographical regions. Supporting this statement, a systematic review and meta-analysis revealed that highest mortality among all co-morbidities was observed in European studies compared to Asian studies despite of lower prevalence of comorbidities (23). In addition, considerable variation in the prevalence of comorbidities and associated severity of disease in different geographical location was observed (23). Other potential reasons, for having not having significant association between co-morbidities with ICU admission are differences in the time of study and variation in the timing of COVID-19 spread. Majority of study were conducted during an early hit of pandemic especially in the elderly population and differences in variant and sub variant of coronavirus might have also created surge in findings. Sample size of the study, prevalence of comorbidities among COVID-19 patients, COVID-19 vaccination, diagnostic testing, reporting accuracy of co-morbidity might have also reported to inconsistent findings. Also the median age of case and control in this study was 59 years with compared to result of one meta-analysis where median age was over 65 years (22). This differences in age might also have role associated with co-morbidity and ICU admission.

Many previous studies found that COVID-19 patients present with lower oxygen saturation, high temperature, pulse rate, respiration rate and blood pressure at the time of hospital admission (17,18,20). These findings also correspond to the present study. Oxygen saturation is the indicator or respiratory functions and demonstrate variations in arterial oxygenation to indicate hypoxemia

in patients. A retrospective study showed that decreased oxygen saturation was significantly associated with ICU admission (17). This was again supported by a nested case- control study conducted in China, patient with SpO2 < 95.5% have 2.35 odds of severe COVID-19 (14). Additionally, study conducted to find out risk factors for clinical deterioration in patients showed that decrease oxygen saturation at emergency department was the independent variable associated with clinical deterioration (3). A cross sectional comparative study conducted in survivors and non- survivors of Nepal also found that non- survivors tended to have lower oxygen saturation at admission (18).

This study also highlights elevated blood pressure at the time of hospital admission was independent risk factor in multivariable analysis. Patients with elevated blood pressure at admission were 9.88 times more likely to be admitted to ICU than normal blood pressure. This finding was consistent with several studies done in China and Denmark where high systolic blood pressure were found to be significantly associated with treatment in ICU (2,24). Furthermore, a study conducted in Colombia to identify the factors associated with admission to ICU also found increased mean arterial pressure on admission significantly associated with patient infected with SARS – CoV- 2 (25).

Pulse rate was found to be significantly associated with ICU admission this finding was consistent with the study done in China where heart rate beats per minute was significantly associated with ICU admission (17). In bivariate analysis, this study found that febrile patients at the time of hospital admission were associated with admission to ICU. Findings was consistent with study done in China in which highest temperature of patients infected with COVID-19 was significant with requirement of ICU care (2). Additionally, Initial body temperature on admission were found significant predictors to patient requiring intensive unit care (16). In this study, respiration rate

was also found significantly associated with admission to ICU. This study showed that patient with tachypnea during the time of hospital admission were 15.5 times more likely to get admitted in ICU. Findings was alike with several studies where respiration rate was found significantly associated with admission to ICU (3,14,17).

Higher leucocyte count indicates our body is fighting infection or inflammation. In this study, total leucocyte count at the time of hospital admission was found to be associated with admission to ICU. Findings are consistent with study conducted in Italy where white blood cells count was significantly associated with risk of death (19). Similar findings was observed from multicenter retrospective case- control study, where WBC was significantly associated with non – survivor group (20). Furthermore, clinical mortality review done in Sukraraj Tropical and Infectious Disease Hospital, among 50 mortality reviewed 80% of patients had lymphocytopenia (26). A contrast finding was concluded by a study done in China where white blood cells were not significantly associated with severity of COVID-19 (14).

C- reactive protein is protein made by liver which is the sign of acute inflammation. In present study CRP was found as significant predictors of ICU admission. Findings was similar with study conducted in China where CRP was found as independent predictors associated with severe COVID-19 (14). Another study done in China also found CRP significantly associated with ICU admission (17). Recent meta-analysis also revealed that most common blood test abnormalities were elevated C- reactive protein(22).

COVID-19 vaccination with either first or second dose with any of available vaccine were not found associated with ICU admission in multivariate analysis. This findings was consistent with study done in central hospital of Nepal showed there was no significant association between vaccination status with outcome of COVID-19 (18). In contrast to this findings, a study done in

England reported vaccination was associated with a significant reduction in symptomatic SARS – CoV2 positive cases (27). The discrepancy in findings might be due to differences in natural immunity between people of different countries.

Present study concluded that patient admitted to ICU had longer hospital stay as compared to patient admitted in COVID-19 isolation ward, which is significantly associated. In this study, median hospital stay days among cases was 13 days and 2 days among control. This findings was consistent with the study conducted in Wuhan where the median time from onset of symptoms to ICU admission was 10.5 days (2). One more study conducted in China showed total in hospital days was 20 days among survivor and 15 days among non – survivor (20). Additionally another study conducted in Nepal, showed median ICU stay days was 10 days in survivors group and 8 days in non – survivor group (18). The possible explanation may be longer duration of stay in ICU due to disease severity, which resulted longer phase of recovery.

Present study found that application of preventive measures was found to be independently associated with ICU admission. Similar findings had been observed in systematic review and meta-analysis where non pharmaceutical Public health interventions showed 16.5% decrease in ICU admission (28). Another study conducted in Thailand also showed wearing masks, handwashing and social distancing were independently associated with lower risk for SARS – CoV-2 infection (29). A systematic review and meta- analysis revealed that several personal protective and social measures including physical distancing, hand washing and wearing masks were associated with reductions in the incidence of COVID -19 (30,31).

This study is of retrospective nature, participants may fail to remember the preventive measures they applied before two weeks of being infected with COVID -19. Thus, it may result in recall

bias. The present study was carried out with small sample size based on a single institution; result may be generalized only to patient under similar setting.

Conclusion

This study concludes that high blood pressure, abnormal C reactive protein, poor preventive practice and longer duration of hospital stay were the predictors associated with ICU admission. This study also identified the risk clinical features and lab investigation during the time of hospital admission. Evaluation of these factors during admission might help for risk assessment and early triage of potential severe COVID-19 cases so that best possible efforts can be made for prompt management of COVID – 19 cases.

Acknowledgement

We would like to acknowledge all participants who allocated time to our study and all people who directly and indirectly supported our study.

References

1. WHO Director-General’s opening remarks at the media briefing on COVID-19 - 11 March 2020 [Internet]. [cited 2022 Mar 2]. Available from: https://www.who.int/director-general/speeches/detail/who-director-general-s-opening-remarks-at-the-media-briefing-on-covid-19---11-march-2020
2. Huang C, Wang Y, Li X, Ren L, Zhao J, Hu Y, et al. Clinical features of patients infected with 2019 novel coronavirus in Wuhan, China. Lancet. 2020;395(10223):497–506.
3. Uranga A, Villanueva A, Lafuente I, González N, Legarreta MJ, Aguirre U, et al. Risk factors for clinical deterioration in patients admitted for COVID-19: A case-control study. Rev Clínica Española (English Ed. 2022;222(1):22–30.
4. Hu Y, Liang W, Liu L, Li L. (No Title). n engl j med. 2020;18:1708–28.
5. Ministry of Health and Population. SitRep Health Sector Response to Novel Coronavirus (2019-nCoV) Confirm Result. 2020;(January):2020.
6. Ministry of Health and Population. SitRep. 2020.
7. Adhikari K, Gautam R, Pokharel A, Dhimal M. Insight into Delta variant dominated second wave of COVID-19 in Nepal. 2020;(January).
8. Ministry of Health and Population. Guidelines for Geriatric Population [Internet]. 2020. Available from: https://drive.google.com/file/d/1K7qNhAkzB2CGvFSIKe8WusjN_edHgCky/view
9. Lancet T. India under COVID-19 lockdown. Lancet [Internet]. 2020 [cited 2022 Mar 2];395:1315. Available from: https://www.
10. WHO. Clinical management Clinical management Living guidance COVID-19. 2021B. 2021;(January):16–44.
11. Interim Clinical Gudance for care of patients with COVID 19 in Healthcare Settings_05-04-2020.pdf.
12. Agarwal A, Ranjan P, Rohilla P, Saikaustubh Y, Sahu A, Dwivedi SN, et al. Development and validation of a questionnaire to assess preventive practices against COVID-19 pandemic in the general population. Prev Med Reports [Internet]. 2021;22(December 2020):101339. Available from: https://doi.org/10.1016/j.pmedr.2021.101339
13. Immovilli P, Morelli N, Antonucci E, Radaelli G, Barbera M, Guidetti D. COVID-19 mortality and ICU admission: The Italian experience. Crit Care. 2020;24:9–11.
14. Li X, Marmar T, Xu Q, Tu J, Yin Y, Tao Q, et al. Predictive indicators of severe COVID-19 independent of comorbidities and advanced age: A nested case -control study. Epidemiol Infect. 2020;
15. Mekonnen S, Id A, Ali SA, Mantfardo B, Basu B. Rate of Intensive Care Unit admission and outcomes among patients with coronavirus: A systematic review and Meta-analysis. 2020; Available from: https://doi.org/10.1371/journal.pone.0235653

16. Heo JN, Han D, Kim HJ, Kim D, Lee YK, Lim D, et al. Prediction of patients requiring intensive care for COVID-19: development and validation of an integer-based score using data from Centers for Disease Control and Prevention of South Korea. J Intensive Care. 2021;9(1):1–9.

17. Zhao Z, Chen A, Hou W, Graham JM, Li H, Richman PS, et al. Prediction model and risk scores of ICU admission and mortality in COVID-19. PLoS One [Internet]. 2020;15(7 July):1–14. Available from: http://dx.doi.org/10.1371/journal.pone.0236618

18. Simkhada N, Pathak BD, Bhusal L, Tandon OP, Shrestha A, Devkota S, et al. Comparison of clinical characteristics and short-term outcomes among COVID-19 patients in a tertiary care center during second wave pandemic in Nepal: a cross-sectional study. F1000Research. 2022;11:903.

19. Ciceri F, Castagna A, Rovere-Querini P, De Cobelli F, Ruggeri A, Galli L, et al. Early predictors of clinical outcomes of COVID-19 outbreak in Milan, Italy. Clin Immunol [Internet]. 2020;217(June):108509. Available from: https://doi.org/10.1016/j.clim.2020.108509

20. Gao J, Zhong L, Wu M, Ji J, Liu Z, Wang C, et al. Risk factors for mortality in critically ill patients with COVID-19: a multicenter retrospective case-control study. BMC Infect Dis. 2021;21(1):1–8.

21. Pathak V, Conklin C. Predictors of ICU Admission and Mortality in Patients with Coronavirus Disease - 2019 (COVID 19) in Community Hospitals. 2021;6:A2551–A2551.

22. Hu J, Wang Y. The Clinical Characteristics and Risk Factors of Severe COVID-19. Gerontology. 2021;67(3):255–66.

23. Thakur B, Dubey P, Benitez J, Torres JP, Reddy S. OPEN A systematic review and meta ‑ analysis of geographic differences in comorbidities and associated severity and mortality among individuals with COVID ‑ 19. Sci Rep [Internet]. 2021;1–13. Available from: https://doi.org/10.1038/s41598-021-88130-w

24. Israelsen SB, Kristiansen KT, Hindsberger B, Ulrik CS, Andersen O, Jensen M. Characteristics of patients with COVID-19 pneumonia at Hvidovre Hospital, March- April 2020. Denish Med J. 2020;(April):28–31.

25. Enrique Machado-Alba JI, Fernando Valladales-Restrepo L, Enrique Machado-Duque M, Gaviria-Mendoza A, Sá nchez-Ramírez N, Felipe Usma-Valencia A, et al. Factors associated with admission to the intensive care unit and mortality in patients with COVID-19, Colombia. 2021; Available from: https://doi.org/10.1371/journal.pone.0260169

26. Bastola, A.; Shrestha, S.; Nepal, R.; Maharjan, K.; Shrestha, B.; Chalise, B.S.; Thapa, P.; Balla, P.; Sapkota A. S. Clinical Mortality Review of COVID-19 Patients at Sukraraj Tropical and Infectious Disease Hospital, Nepal; A retrospective Study. Trop Med Infect Dis. 2021;

27. Bernal JL, Andrews N, Gower C, Stowe J, Robertson C, Tessier E, et al. Early effectiveness of COVID-19 vaccination with BNT162b2 mRNA vaccine and ChAdOx1 adenovirus vector vaccine on symptomatic disease, hospitalisations and mortality in older

adults in England. medRxiv [Internet]. 2021;2021.03.01.21252652. Available from: https://doi.org/10.1101/2021.03.01.21252652

28. 1☯ SI, Gholipour K, 2☯ ID, Azami-Aghdash S, Ghiasi A, Rezapour A, et al. Effectiveness of non-pharmaceutical public health interventions against COVID-19: A systematic review and meta-analysis. 2021;

29. Doung-Ngern P, Suphanchaimat R, Panjangampatthana A, Janekrongtham C, Ruampoom D, Daochaeng N, et al. Case-Control Study of Use of Personal Protective Measures and Risk for SARS-CoV 2 Infection, Thailand. Emerg Infect Dis. 2020;26(11):2607–16.

30. Talic S, Shah S, Wild H, Gasevic D, Maharaj A, Ademi Z, et al. Effectiveness of public health measures in reducing the incidence of covid-19, SARS-CoV-2 transmission, and covid-19 mortality: Systematic review and meta-analysis. BMJ. 2021;375:1–15.

31. Regmi K, Lwin CM. Factors associated with the implementation of non-pharmaceutical interventions for reducing coronavirus disease 2019 (Covid-19): A systematic review. Int J Environ Res Public Health. 2021;18(8):1–27.

Annexure - I

Questionnaire to assess prevention practices against COVID-19 in the general population

Kindly fill the questionnaire below based on your routine for last two weeks.

SN	Item	Options				
1	How often do you shake hands while greeting people nowadays?	Always (more than 90% times)	Mostly (Approx 75% times)	Commonly (Approx 50% times)	Occasionally (approx. 25% times)	Rarely (less than 10% times)
2	How often do you wash/sanitize your hands with soap and water/alcohol-based sanitizer?	Always (more than 90% times)	Mostly (Approx 75% times)	Commonly (Approx 50% times)	Occasionally (approx. 25% times)	Rarely (less than 10% times)
3	How often do you ensure that you wash/sanitize your hands for at least 20s?	Always (more than 90% times)	Mostly (Approx 75% times)	Commonly (Approx 50% times)	Occasionally (approx. 25% times)	Rarely (less than 10% times)
4	How often do you ensure that you cover your face with a handkerchief/bent elbow while coughing/sneezing?	Always (more than 90% times)	Mostly (Approx 75% times)	Commonly (Approx 50% times)	Occasionally (approx. 25% times)	Rarely (less than 10% times)
5	How often do you ensure that you clean your hands before touching your eyes/nose/mouth?	Always (more than 90% times)	Mostly (Approx 75% times)	Commonly (Approx 50% times)	Occasionally (approx. 25% times)	Rarely (less than 10% times)
6	How often do you maintain a minimum distance of one meter at your workplace?	Always (more than 90% times)	Mostly (Approx 75% times)	Commonly (Approx 50% times)	Occasionally (approx. 25% times)	Rarely (less than 10% times)

7	How often do you maintain a minimum distance of one meter while eating food with your colleagues at your workplace?	Always (more than 90% times)	Mostly (Approx 75% times)	Commonly (Approx 50% times)	Occasionally (approx. 25% times)	Rarely (less than 10% times)
8	How often do you avoid going out of the house unnecessarily?	Always (more than 90% times)	Mostly (Approx 75% times)	Commonly (Approx 50% times)	Occasionally (approx. 25% times)	Rarely (less than 10% times)
9	How often have you attended social gathering in the past two months? (Like meeting friends, going to religious places, visiting malls, theaters etc)	Never (5)	Once (4)	Twice (3)	Thrice (2)	More than three times (1)
10	How often do you maintain distance of one meter in public spaces (eg grocery shopping, social gathering etc) ?	Always (more than 90% times)	Mostly (Approx 75% times)	Commonly (Approx 50% times)	Occasionally (approx. 25% times)	Rarely (less than 10% times)
11	How often do you wear masks while going out of home?	Always (more than 90% times)	Mostly (Approx 75% times)	Commonly (Approx 50% times)	Occasionally (approx. 25% times)	Rarely (less than 10% times)
12	While wearing a mask, how often do you ensure that both your nose and mouth are covered?	Always (more than 90% times)	Mostly (Approx 75% times)	Commonly (Approx 50% times)	Occasionally (approx. 25% times)	Rarely (less than 10% times)
13	How often do you keep your mask properly in a separate bag/dustbin after using it?	Always (more than 90% times)	Mostly (Approx 75% times)	Commonly (Approx 50% times)	Occasionally (approx. 25% times)	Rarely (less than 10% times)
14	How often do you sanitize your personal items (eg purse/mobile phone, etc) with sanitizer when you come home?	Always (more than 90% times)	Mostly (Approx 75% times)	Commonly (Approx 50% times)	Occasionally (approx. 25% times)	Rarely (less than 10% times)
15	How often do you take precautions when buying things to avoid virus contamination?	Always (more than 90% times)	Mostly (Approx 75% times)	Commonly (Approx 50% times)	Occasionally (approx. 25% times)	Rarely (less than 10% times)

16	How often do you obey government restrictions regarding the COVID-19 pandemic?	Always (more than 90% times)	Mostly (Approx 75% times)	Commonly (Approx 50% times)	Occasionally (approx. 25% times)	Rarely (less than 10% times)
17	In case you develop symptoms of the disease, you will contact the hospital/helpline/authority regarding it?	Strongly agree	Agree	Can't say	Disagree	Strongly disagree
18	If you come in contact with COVID positive/suspect person, you would stop going to work and confine yourself to the home away from friends and family members.	Strongly agree	Agree	Can't say	Disagree	Strongly disagree

Annexure – II

Nepali version of Preventive practice questionnaire

1. आजकल तपाई मानिसहरूलाई अभिवादन गर्दा कति पटक हात मिलाउनुहुन्छ?
   - सधैं)९०% भन्दा बढी पटक(
   - प्रायः)लगभग ७५% पटक(
   - सामान्यतया)लगभग ५०% पटक(
   - कहिलेकाहीँ)लगभग २५% पटक(
   - विरलै)१०% पटक भन्दा कम(

2. तपाइँ कति पटक साबुन र पानी/अल्कोहलमा आधारित सेनिटाइजरले तपाइँको हात धुनुहुन्छ / सेनिटाइज गर्नुहुन्छ ?
   - सधैं)९०% भन्दा बढी पटक(
   - प्रायः)लगभग ७५% पटक(
   - सामान्यतया)लगभग ५०% पटक(
   - कहिलेकाहीँ)लगभग २५% पटक(
   - विरलै)१०% पटक भन्दा कम(

3. तपाईंले कम्तिमा 20 सेकेन्डसम्म आफ्नो हात धुने/सनिटाइज गर्ने कुरा कति पटक सुनिश्चित गर्नुहुन्छ?
   - सधैं)९०% भन्दा बढी पटक(
   - प्रायः)लगभग ७५% पटक(
   - सामान्यतया)लगभग ५०% पटक(
   - कहिलेकाहीँ)लगभग २५% पटक(

- विरलै)१०% पटक भन्दा कम(

4. तपाईंले खोक्दा/हाछ्युँ गर्दा कति पटक यो सुनिश्चित गर्नुहुन्छ कि तपाईंले आफ्नो अनुहार रुमाल/ कुहिनोले छोप्नु हुन्छ ?
   - सधैं)९०% भन्दा बढी पटक(
   - प्रायः)लगभग ७५% पटक(
   - सामान्यतया)लगभग ५०% पटक(
   - कहिलेकाहीँ)लगभग २५% पटक(
   - विरलै)१०% पटक भन्दा कम(

5. तपाइँ कति पटक तपाइँको आँखा / नाक / मुख छुनु अघि तपाइँको हातहरू सफा छ/छैन भनेर सुनिश्चित गर्नुहुन्छ ?
   - सधैं)९०% भन्दा बढी पटक(
   - प्रायः)लगभग ७५% पटक(
   - सामान्यतया)लगभग ५०% पटक(
   - कहिलेकाहीँ)लगभग २५% पटक(
   - विरलै)१०% पटक भन्दा कम(

6. तपाईं आफ्नो कार्यस्थलमा न्यूनतम एक मिटरको दुरी कति पटक कायम गर्नुहुन्छ?
   - सधैं)९०% भन्दा बढी पटक(
   - प्रायः)लगभग ७५% पटक(
   - सामान्यतया)लगभग ५०% पटक(
   - कहिलेकाहीँ)लगभग २५% पटक(
   - विरलै)१०% पटक भन्दा कम(

7. तपाईको कार्यस्थलमा आफ्ना सहकर्मीहरूसंग खाना खाँदा कति पटक न्यूनतम एक मिटरको दुरी कायम राख्नुहुन्छ ?
   - सधैं)९०% भन्दा बढी पटक(
   - प्रायः)लगभग ७५% पटक(
   - सामान्यतया)लगभग ५०% पटक(
   - कहिलेकाहीँ)लगभग २५% पटक(
   - विरलै)१०% पटक भन्दा कम(

8. अनावश्यक रूपमा घरबाहिर ननिस्कन कत्तिको बेवास्ता गर्नुहुन्छ ?

- सधैं)९०% भन्दा बढी पटक(
- प्रायः)लगभग ७५% पटक(
- सामान्यतया)लगभग ५०% पटक(
- कहिलेकाहीँ)लगभग २५% पटक(
- विरलै)१०% पटक भन्दा कम(

9. विगत दुई महिनामा तपाईले कति पटक सामाजिक जमघटमा भाग लिनुभयो? (जस्तै बैठक, साथीहरू भेट्ने , धार्मिक स्थलहरूमा जाने, मल, थिएटरहरू, आदि)

- कहिले पनि छैन
- एक चोटी
- दुई चोटी
- तिन चोटी
- तिन भन्दा धेरै

10. तपाईले कति पटक सार्वजनिक स्थानहरूमा न्यूनतम एक मिटरको दूरी कायम गर्नुहुन्छ)जस्तै किराना किनमेल, सामाजिक जमघट, आदि(?
    - सधैं)९०% भन्दा बढी पटक(
    - प्रायः)लगभग ७५% पटक(
    - सामान्यतया)लगभग ५०% पटक(
    - कहिलेकाहीँ)लगभग २५% पटक(
    - विरलै)१०% पटक भन्दा कम(

11. घरबाहिर निस्कँदा कति पटक मास्क लगाउनुहुन्छ ?
    - सधैं)९०% भन्दा बढी पटक(
    - प्रायः)लगभग ७५% पटक(
    - सामान्यतया)लगभग ५०% पटक(
    - कहिलेकाहीँ)लगभग २५% पटक(
    - विरलै)१०% पटक भन्दा कम(

12. मास्क लगाउँदा, तपाइँ कति पटक यो सुनिश्चित गर्नुहुन्छ कि तपाइँको नाक र मुख दुबै छ ढाकिएको छ ?
    - सधैं)९०% भन्दा बढी पटक(
    - प्रायः)लगभग ७५% पटक(

- सामान्यतया)लगभग ५०% पटक(
- कहिलेकाहीँ)लगभग २५% पटक(
- विरलै)१०% पटक भन्दा कम(

13. तपाईंले कति पटक आफ्नो मास्क प्रयोग गरिसकेपछि अलग्गै झोला/डस्टबिनमा राम्ररी राख्नुहुन्छ?

- सधैं)९० % भन्दा बढी पटक(
- प्रायः)लगभग ७५% पटक(
- सामान्यतया)लगभग ५०% पटक(
- कहिलेकाहीँ)लगभग २५% पटक(
- विरलै)१०% पटक भन्दा कम(

14. तपाई घर आइसके पछि आफ्नो व्यक्तिगत वस्तुहरू)जस्तै पर्स/मोबाइल फोन, इत्यादि(कति पटक सेनिटाइजरले सेनिटाइज गर्नुहुन्छ ?

- सधैं)९०% भन्दा बढी पटक(
- प्रायः)लगभग ७५% पटक(
- सामान्यतया)लगभग ५०% पटक(
- कहिलेकाहीँ)लगभग २५% पटक(
- विरलै)१०% पटक भन्दा कम(

15. सामान किन्दा भाइरस संक्रमणबाट बच्न कति पटक सावधानी अपनाउनुहुन्छ?

- सधैं)९०% भन्दा बढी पटक(
- प्रायः)लगभग ७५% पटक(
- सामान्यतया)लगभग ५०% पटक(
- कहिलेकाहीँ)लगभग २५% पटक(
- विरलै)१०% पटक भन्दा कम(

16. कोभिड-१९ महामारीको सन्दर्भमा सरकारी प्रतिबन्धहरूको कत्तिको पालना गर्नुहुन्छ?

- सधैं)९०% भन्दा बढी पटक(
- प्रायः)लगभग ७५% पटक(
- सामान्यतया)लगभग ५०% पटक(
- कहिलेकाहीँ)लगभग २५% पटक(
- विरलै)१०% पटक भन्दा कम(

17. यदि तपाईंलाई रोगको लक्षणहरू देखा परेमा, तपाईंले यस सम्बन्धमा अस्पताल/हेल्पलाइन/ अख्तियार मा सम्पर्क गर्नुहुनेछ ?

- एकदमै सहमत

- सहमत
- भन्न सक्दिन
- असहमत
- एकदमै असहमत

18. दि तपाइँ कोभिड पोजिटिभ/शङ्कास्पद व्यक्तिको सम्पर्कमा आउनुभयो भने, तपाइँ काममा जान बन्द गरि आफूलाई साथीहरू र परिवारका सदस्यहरूबाट टाढा घरमा सीमित राख्नु हुन्छ ?
   - एकदमै सहमत
   - सहमत
   - भन्न सक्दिन
   - असहमत
   - एकदमै असहमत
     - अन्य कारणहरू)कृपया निर्दिष्ट गर्नुहोस्(:

Letter of data collection

Click here to access/download
Supporting Information
supporting letter.jpg

Figure

Click here to access/download
Supporting Information
patient enrollment flowchart.tif

Click here to access/download
Supporting Information
Table 1.tif

Click here to access/download
Supporting Information
Table 1 continue.tif

Click here to access/download
Supporting Information
Table 2.tif

Click here to access/download
Supporting Information
Table 3.tif

Click here to access/download
Supporting Information
Table 4.tif

Click here to access/download
Supporting Information
Table 5.tif

Click here to access/download
Supporting Information
Table 6.tif

Formatted: Numbering: Continuous

PREDICTORS TO INTENSIVE CARE UNIT ADMISSION AMONG PATIENT WITH CORONAVIRUS DISEASE IN SUKRARAJ TROPICAL AND INFECTIOUS DISEASE HOSPITAL, NEPAL: A CASE CONTROL STUDY

Dipsikha Aryal[1], Paras Kumar Pokharel[1], Anup Ghimire[1], Vijay Kumar Khanal[1], Gyanu Nepal Gurung[1], Bimal Sharma Chalise[2], Sudikshya Neupane[1], Shikha Basnet[1]

Affiliations

[1] B.P Koirala Institute of Health Sciences, School of Public Health and Community Medicine, Dharan, Nepal

[2] Sukraraj Tropical and Infectious Disease Hospital, Teku, Nepal

Corresponding author: *Dipsikha Aryal*

Phone number: +977- 9840143527

Email ID: seekuary@gmail.com

ABSTRACT

Introduction

The clinical features of COVID-19 are varied, ranging from asymptomatic states or mild upper respiratory tract infections to severe pneumonia with respiratory failure acute respiratory distress syndrome in the early stages, or even death. Studies have shown that 20.0% of patients require hospitalization with 10.0–20.0% being admitted to the intensive care unit. This study aimed to assess predictors associated with COVID-19 leading to ICU admission among reverse transcriptase- polymerase chain reaction positive patients in Sukraraj Tropical and infectious disease hospital, Nepal.

Methods

We conducted a case-control study from June 2022 to July 2022 among patients admitted to Sukraraj Tropical and Infectious Disease Hospital. Hospital-based age (± 2 years) and sex-matched case-control study design was adopted in which ICU admitted (case group, n= 33) and General ward admitted (control group, n=66) were taken. We collected data through a review of hospital files and telephone interviews using a structured questionnaire consisting of socio-demographics, clinical and preventive predictors. Data were analyzed using the statistical package for social science version 11.5. We present parametric numerical variables as mean and standard deviation, and categorical variables as percentage and frequency. We applied the Chi-square test and binary conditional logistics regression to determine the predictors associated with ICU admission.

Results

High blood pressure, high C-reactive protein,and -poor application of preventive practice and longer duration of hospital stay were found to be the predictors of ICU admission. The adjusted

Formatted: Strikethrough

~~multivariable~~ conditional logistics regression analyses revealed that independent risk factors associated with ICU admission were ~~high~~ elevated blood pressure (AOR = 2.22; 95% CI 1.05 – 4.71, p 2.2= 0.015), abnormal CRP (AOR = 2.92; 95% CI 1.24 – 6.84, p = 0.012) at the time of hospital admission~~-~~ were more likely to get admitted to ICU. Patient with poor preventive practice (AOR = 3.34; 95% CI 1.19 – 9.31, p=0.02) more likely to get admitted to ICU than patient with good preventive practice.

~~(AOR = 9.88; 95% CI: 1.4 – 68.90, p= 0.021)~~ ~~, high C reactive protein (AOR = 12.23; 95% CI: 1.37 – 108.53, p= 0.025), poor preventive practice (AOR= 16.22; 95% CI: 2.26 – 116.40, p= 0.006), duration of hospital stay (AOR = 1.45; 95% CI: 1.17 – 1.79, p= 0.001).~~

Conclusion: These findings could be utilized for early triage and risk assessment in COVID-19 patients. Developing evidence-based and appropriate community-based preventive practice awareness programs among the general public is recommended.

Keywords: COVID-19, Intensive care unit, Preventive practices

INTRODUCTION

Coronavirus disease 2019 (COVID-19) caused by the severe acute respiratory syndrome coronavirus 2 (SARS-CoV-2) previously known as 2019-nCoV which was first identified in Wuhan China in December 2019 and World Health Organization declared a pandemic in March 2020 (1).

The clinical features of COVID-19 are varied, ranging from asymptomatic states or mild upper respiratory tract infections to severe pneumonia with respiratory failure acute respiratory distress syndrome in the early stages or even death. Studies have shown that major symptoms ~~like~~ such as fever, cough, dyspnea and minor symptoms such as loss of taste and smell, gastrointestinal symptoms and cutaneous manifestations are common clinical features of this disease (2).

The majority of patients progress favorably. However, 20% of patients require hospitalization and their disease can progress rapidly to acute respiratory distress syndrome (ARDS), multiple organ dysfunction syndrome, or even death (3). Rates for the cases requiring intensive care are low, with 10–20% being admitted to the intensive care unit (ICU), 3–10% requiring intubation, and 2–5% dead. Severe patients show respiratory failure, pneumonia, multi-organ failure, and shock, which require care in intensive care facilities (4).

The first case of coronavirus was ~~seen~~ observed in a Nepalese Citizen returning from Wuhan City returned on 9 January 2020 and visited Sukraraj Tropical and Infectious Disease Hospital on 13 January 2020. National Public Health Laboratory (NPHL) communicated the positive test result for 2019-nCoV on 23 January 2020 (5). On 23rd March 2020, a second case of COVID-19 was seen in Nepal. Immediately after the confirmation of the second case nationwide lockdown was ~~endorsed in Nepal from 25th March 2020~~ endorsed nationwide in Nepal on March 25, 2020 (6). Studies show the basic reproductive number during the second wave was larger than the first wave

(7). The expected cases from September 2021 to April 2022 were 111,300 among which 3590 need ICU (7).

A variety of containment and mitigation strategies have been adopted in several countries to adequately respond to covid-19, with the intention of deferring major surges of patients in hospitals and protecting the most vulnerable people from infection, COVID-19 clinical management guidelines, guidelines for the use of personal protective equipment, guidelines for waste management including elderly people and those with comorbidities (8). Several factors like diverse cultures, wide socioeconomic disparities and healthcare services inequalities might pose challenges in the implementation of these preventive practices at the community level (9).

In Nepal, to date, most clinical studies on COVID-19 have focused on describing the general epidemiologic and clinical characteristics. Studies are mainly focused on comparing the association between survivor and non – survivor group, RT PCR for COVID-19 test positive and negative group but data on comparison between ICU admitted and non-admitted group is still lacking. However, combined data on how the risks associated with demographic, underlying co-morbidities, clinical and public health predictors in this setting is limited. In this context, we conducted a study to assess predictors associated with COVID-19 leading to ICU admission among RT PCR positive for SARS-COV-2 patients in Sukraraj Tropical and Infectious Disease Hospital.

MATERIALS AND METHODS

Study Design, population and setting

We conducted a hospital-based (age +/- 2 years and sex) matched case-control study, where proposal writing started from 1st April 2022, followed by data collection from 24th June 2022 to 12th July 2022 among Reverse transcriptase polymerase chain reaction positive for SARS-COV-2 patients admitted to covid ICU and general ward of Sukraraj Tropical and Infectious Disease Hospital.

Study site

Formatted: Font: Times New Roman, 12 pt, Bold

According to MoHP, latest updates on 6 August 2021 there are 78 government hospitals operating COVID-19 care units. In Kathmandu valley, there are 10 public hospitals with High dependency unit (HDU), Intensive care unit (ICU) and ventilators. Among them, Sukraraj tropical and infectious disease was selected for this study, which is only central government infectious disease hospital of Nepal and fully dedicated COVID hospital. This hospital is only one infectious disease hospital in Nepal with 26 general beds, 50 HDU beds, 24 ICU beds with 22 ventilators which is used along with ICU beds. Sukraraj tropical and infectious disease hospital is also one of the certified laboratory for RT- PCR and provide COVID-19 care along with ICU facility and most trustable hospital of general public.

Formatted: Font: Times New Roman, 12 pt

Sample size

We calculated the sample size for this study by taking reference to case-control study done in Spain on 2021 (3), the proportion of cases (p_1) is 33.33% and the proportion of control (p_2) was 66.67%. Taking a 95% confidence interval and 80% power of test by using the formula of two proportion the calculated sample size was 33. A sample of 33 cases and 66 control was chosen in (1:2 ratio), the total number of samples was 99.

Sampling technique

Sukraraj Tropical and Infectious Disease Hospital was selected from the list of public hospital with a High Dependency Unit, Intensive Care Unit and ventilators in Kathmandu Valley. A list of COVID-19 RT-PCR positive patients admitted to ICU and general ward (Covid ward/ Covid isolation ward) in Sukraraj Tropical and Infectious Disease Hospital from Shrawan to Poush 2078 BS (16th July 2021 – 14th January 2022) was collected. The complete frame of ICU admitted patients which was 222 during six months was divided by the calculated sample size for this study which was 99. Each 2.24 ~ 3rd ICU admitted patient file was reviewed, that baseline characteristics, oxygen saturation, blood pressure, pulse rate, respiration rate and temperature at the time of hospital admission and laboratory electronic record Total Leucocyte Count (TLC), C-Reactive Protein (CRP) and Cycle threshold value (CT value) were recorded. Systematic random sampling was used for the selection of cases, if the selected 3rd respondents did not meet the inclusion criteria, then the next file was reviewed.

For a selection of control, COVID-19 RT-PCR positive control having at least one or more symptoms during the clinical course of the disease and admitted in general ward was selected according to age and sex-matched with the case. For collecting data on preventive measures, the patient was contacted via phone call.

Among 713 total COVID-19 patients admitted during six months, 2.1% of participants were not eligible for the study because of age limitation. Out of 698, 222 were admitted to ICU and 371 were admitted to COVID-19 general ward. Among 698, 105 (15.04%) were excluded due to death during disease treatment. Out of 593, 143 files were reviewed and data was recorded and each participant was contacted. Among 143 contacted, 2 (1.39%) were excluded due to death after discharge from the hospital. From the remaining 141 participants, 33 cases were matched with 66 control and the remaining 42 unmatched participants were also excluded from this study.

Figure 1 Flowchart of patient enrollment

Populations / Participants selection criteria

Formatted: Font: Times New Roman, 12 pt

Inclusion criteria for cases

Reverse transcriptase polymerase chain reaction positive for SARS-COV-2, admitted to COVID ICU of Sukraraj tropical and infectious disease hospital during the clinical course of disease from Shrawn to Poush 2078, patient who recovered, recovered with sequelae, referred, left against medical advice after ICU admission, adult > 18 years of age and for questions related to preventive practice those who gave verbal consent was included in this study.

For control

Reverse transcriptase polymerase chain reaction positive for SARS-COV-2, admitted to covid ward, isolation ward, cabin of Sukraraj tropical and infectious disease hospital, patient who recovered, recovered with sequelae, referred, left against medical advice after admission, had at least one or more subjective experience of symptoms during clinical course of disease, adult >18 years, and for questions related to preventive practice those who gave verbal consent was included in this study.

Exclusion criteria for cases and control

Reverse transcriptase polymerase chain reaction positive for SARS-COV-2 but not admitted to Sukraraj tropical and infectious disease hospital, patient who deceased during and after ICU and COVID ward admission, age < 18 years, COVID-19 Positive test report identified by any other rapid diagnostic test, pregnant women, severely-ill patients and cognitive impairment (not able to identify year, month, district, their age, year of birth or address) and who did not give verbal consent was excluded from study.

Ethical approval

We obtained Ethical clearance from the Institutional ethical review committee of B.P. Koirala Institute of Health Sciences (Reference number: 236/078/079-IRC) before conducting the study. Study permission was taken from Sukraraj Tropical and Infectious Disease Hospital (Reference number: 078/79 444) before commencing the study. Due to retrospective nature of study, it was

not possible to visit each respondent, therefore verbal consent was taken from the respondents before enrolling them in the study. The confidentiality and anonymity of the participants were maintained and assured throughout the study.

Data collection

Data was collected in a retrospective way using a structured questionnaire that consists of three sections- a) Socio-demographics characteristics; b) Clinical predictors; c) Preventive predictors.

Assessment of socio-demographic and clinical Predictors

Clinical data at hospital admission, including demographic information, chronic comorbidities, vital signs, symptoms, diagnosis of severity of COVID-19, date of admission, date of discharge, COVID-19 vaccination status and outcomes were extracted from medical records using a data collection form.

The severity at admission was categorized into four groups: mild, moderate, severe, and critical, based on the World Health Organization (WHO) COVID-19 Clinical Management Guidelines (Living Guidance 25 January 2021) (10,11).

Lab parameters including threshold cycle CT value, Total Leucocyte Count and C-reactive protein were recorded from an electronic record of a laboratory.

Assessment of preventive predictors

A telephone interview was conducted with each case and control for preventive measures that the patient performed before two weeks of COVID-19 infection. Furthermore, to ascertain the socio-

demographics, epidemiological and symptoms data, which were not available from medical records the researcher directly communicated with patients or their families. Other questions include the following components:

Marital status, ethnicity, religion, occupation, family type, family size, means of transportation, smoking and alcohol habit. For preventive practice, a “Questionnaire to assess prevention practice against COVID-19 in general population” was used which was developed by AIIMS New Delhi India.

Description of Preventive practice questionnaire

“Questionnaire to assess prevention practice against COVID-19 in general population” Was developed by AIIMS New Delhi India (12). (Please refer to Annexure – I and Annexure – II for the Questionnaire form in English and Nepali). Preventive practice against COVID-19 pandemic in general population consists of two sections of questions, where the first section assess the preventive practice and the second section assesses the reasons for reasons why preventive practice isn’t being followed. Section A consists of 18 questions where question 1 is related to hand, 2, 3 and 5 are related with hand hygiene, question 6, 7 and 10 are related to social distancing, questions 11-13 are related with mask use, questions 4, 14 and 15 are related with basic precautions, questions 8,9, 16 are related with safety and following government restrictions and 17, 18 are related with health care behavior. For this study, only section A of the questionnaire was used.

Statistical analysis

Data was collected in a paper-based questionnaire which was entered in Statistical Package for Social Sciences (SPSS) 11.5 version. Case-control matching was performed in SPSS. We present

parametric numerical variables as mean and standard deviation, and categorical variables as percentage and frequency. The Kolmogorov – Smirnov test and Shapiro – Wilk test was conducted to evaluate whether the data were normally distributed. We applied the Chi-square test for categorical variables and Mann- Whitney U test to compare the association between numerical variables with ICU admission. ~~Binary~~ Conditional logistics regression was done to determine the predictors associated with ICU admission. We presented the results of ~~binary~~ conditional logistic regression using adjusted odds ratios (AOR), their 95% confidence intervals and p-values. We conducted all tests at a 95% confidence level and considered p-values less than 0.05 to be statistically significant.

Formatted: Strikethrough

Formatted: Strikethrough

Results

A total of 99 patients were randomly selected from 713 patients who were hospitalized during the study period in participating centers. Table 1 shows the median age of cases was 59 (42 – 70) years, while the median age of control was 59 (43.25 – 70.00) years. Among married, 35.7% were cases and 64.3% of control. Current residence in municipality (38.5% vs 61.5%, p= 0.255), secondary education (37.5% vs 62.5%, p=0.104), unemployed (34.1% vs 65.9%, p=0.138). Among Brahmin/ Chhetri, 31.0% were case and 69.0% were control. Hindu accounts for 34.1% cases and 65.9% control. Among nuclear family, 40.0% were case and 60.0% control. socio-demographic variables were not found to be significantly associated with ICU admission.

Table 1: Association between sociodemographic characteristics with ICU admission

Formatted: Font: Italic

~~**[a] = Pearson chi square**~~
~~**[b] = Fischer’s exact test**~~
~~**[c] = Linear by linear chi square**~~

Table 2 shows the association between co-morbidities and behavioural characteristics with ICU admission. Pre-existing co -morbidities including diabetes, hypertension, cardiac disease, COPD and other medical issues were not significantly associated with ICU admission. In behavioural characteristics, smoking was found to be significantly associated ($p = 0.005$) with ICU admission. Smoker was 3.4 (95% CI: 1.4 – 8.3) times likely admitted in ICU than those who were non-smoker.

Table 2: Association between Co-morbidities and behavioral characteristics with ICU admission

Variables	ICU admission (Cases) n (%)	General admission (Control) n (%)	χ2 test p value	Crude odd's ratio (95% CI)
**Pre-existing Co-morbid illness**				
No	14 (25.9)	40 (74.1)	0.087 [a]	2.08 (0.89 – 4.8)
Yes	19 (42.2)	26 (57.8)		
**Diabetes Mellitus**				
No	28 (32.6)	58 (67.4)	0.674 [a]	0.77 (0.23 – 2.57)
Yes	5 (38.5)	8 (61.5)		
**Hypertension**				
No	22 (31.4)	48 (68.6)	0.532 [a]	
Yes	11 (37.9)	18 (62.1)		0.75 (0.30 – 1.85)
**Cardiac disease**				
No	31 (32.3)	65 (67.7)	0.257 [b]	0.23 (0.02 – 2.7)
Yes	2 (66.7)	1 (33.3)		
**COPD**				
No	26 (30.2)	60 (69.8)	0.092 [a]	0.37 (0.11 – 1.21)
Yes	7 (53.8)	6 (46.2)		
**Other medical issues**				
No	31 (33.7)	61 (66.3)	0.782 [b]	0.78 (0.14 – 4.29)
Yes	2 (28.6)	5 (71.4)		
**Smoking**				
Non- smoker	15 (23.4)	49 (76.6)	**0.005** [a]	3.4 (1.4 – 8.3)
Smoker	18 (51.4)	17 (48.6)		
**Alcohol**				
Non- Alcoholic	17 (29.8)	40 (70.2)	0.388 [a]	1.4 (0.62 – 3.36)
Alcoholic	16 (38.1)	26 (61.9)		

Formatted: Font: Italic

Formatted Table

[a] = Pearson chi- square

[b] = Fischer's exact test

Table 3 shows the severity of COVID-19 during the time of hospital admission. Table shows variables were significantly associated with ICU admission.

Table 3: Severity of COVID-19 at the time of hospital admission

Variables	ICU admission (Cases) n (%)	General admission (Control) n (%)	$\chi 2$ test p value
Mild COVID -19	0 (0.0)	31 (100.0)	
Moderate COVID -19 infection	0 (0.0)	7 (100.0)	0.001
Moderate COVID -19 pneumonia	0 (0.0)	8 (100.0)	
Severe COVID -19 pneumonia	33 (62.3)	20 (37.7)	

Table 4 shows the association of clinical characteristics, lab parameter and COVID-19 vaccination with ICU admission. The odds of ICU admission were 20.0 (95% CI: 5.4 – 72.83; p value: <0.001) times higher in patient with low oxygen saturation than normal oxygen. Patients with elevated blood pressure had 6.10 (95% CI: 2.40 – 15.50; p value: <0.001) odds of ICU admission as compared to normal blood pressure. Patients with pulse rate above 100 bpm had 7.75 (95% CI: 2.23 - 26.87; p value: <0.001) times higher odds of ICU admission as compared to normal pulse rate. Febrile patient had 14.44 (95% CI: 1.65 - 125.75; p value: 0.005) chances of ICU admission

Commented [O1]: Table added after comment

Formatted: Font: Italic

in comparison with afebrile patients. Patients with tachypnea had 15.5 (95% CI: 3.4 – 70.10; p value: <0.001) higher odds of ICU admission in comparison with normal respiration rate.

Patients with abnormal Total Leucocyte Count had 10.07 (95% CI: 2.94 – 34.4; p value: <0.001) more chance of ICU admission in contrast with normal TLC. Patients with abnormal CRP had 7.18 (95% CI: 2.77 – 18.65; p value: <0.001) chances of ICU admission in comparison with normal CRP.

Patients without COVID-19 vaccination had 6.78 (95% CI: 2.63 – 17.48; p value: <0.001) higher chance of ICU admission in contrast to those vaccinated either first or second dose with any kind of vaccine.

274 ***Table 4:*** *Association between clinical and lab parameter with ICU admission*

Formatted: Font: Italic

Variables	ICU admission (Case) (n=33) N %	General admission (Control) (n=66) N %	χ2 test p value	Odds Ratio	95% CI (Lower-Upper)
**Oxygen saturation**					
Normal*	3 (6.4)	44 (93.6)	**<0.001***	20.00	5.4 - 72.83
Below	30 (57.7)	22 (42.3)			
**Blood Pressure**					
Normal*	14 (20.6)	54 (79.4)	**<0.001***	6.10	2.40 – 15.50
Elevated	19 (61.3)	12 (38.7)			
**Pulse rate**					
Normal*	22 (26.2)	62 (73.8)	**<0.001***	7.75	2.23 - 26.87
Above	11 (73.3)	4 (26.7)			
**Temperature**					
Afebrile*	27 (29.3)	65 (70.7)	**0.005***	14.44	1.65 - 125.75
Febrile	6 (85.7)	1 (14.3)			
**Respiration rate**					
Normal*	2 (5.7)	33 (94.3)	**<0.001***	15.5	3.4 – 70.10
Tachypnea	31 (48.4)	33 (51.6)			
**Total leucocyte count**					
Normal*	20 (24.4)	62 (75.6)	**<0.001***	10.07	2.94 – 34.4
Abnormal	13 (76.5)	4 (23.5)			
**C-reactive protein**					
Normal*	8 (14.8)	46 (85.2)	**<0.001***	7.18	2.77 – 18.65
Abnormal	25 (55.6)	20 (44.4)			
**COVID-19 vaccination**					
Yes*	14 (20.3)	55 (79.7)	**<0.001***	6.78	2.63 – 17.48
No	19 (63.3)	11 (36.7)			

*Reference category

Table 5 shows association between preventive practice and ICU admission. This table explains that patient who performed poor preventive practice had 12.97 times higher odds of getting admitted to ICU in comparison with good preventive practice. Good preventive practice was described as the total score above 54 which was the average score among total.

***Table 5:** Association between preventive practice and ICU admission*

Variables	ICU admission Case (n=33) n %	General admission Control (n=66) n %	p-value	Odds Ratio	95% CI (Lower-Upper)
**Preventive practice**					
Good practice*	6 (18.2%)	49 (74.2%)	< 0.001*	12.97	4.57 – 36.79
Poor practice	27 (81.8%)	17 (25.8%)			

* Reference

Table 6 shows, variables that were significantly associated with ICU admission, with a p-value less than or equal to 0.2 in bivariate analysis were taken for multivariate logistics regression analysis. Variables with expected count less than 5 were not included in multivariate logistics regression analysis.

The adjusted multivariable logistics regression analyses revealed that independent risk factors associated with ICU admission were high blood pressure (AOR = 9.88; 95% CI: 1.4 – 68.90, p= 0.021), high C reactive protein (AOR = 12.23; 95% CI: 1.37 – 108.53, p= 0.025), poor preventive practice (AOR= 16.22; 95% CI: 2.26 – 116.40, p= 0.006), duration of hospital stay (AOR = 1.45; 95% CI: 1.17 – 1.79, p= 0.001).

Formatted: Font: Italic

Formatted: Strikethrough

Table 6 shows the results of conditional logistics regression. Patients with elevated Blood pressure (AOR = 2.22; 95% CI 1.05 – 4.71, p = 0.015), Abnormal CRP (AOR = 2.92; 95% CI 1.24 – 6.84, p = 0.012) at the time of hospital admission were more likely to get admitted to ICU. Patient with poor preventive practice (AOR = 3.34; 95% CI 1.19 – 9.31, p=0.02) more likely to get admitted to ICU than patient with good preventive practice.

Formatted: Font: Italic

Table 6: *Conditional logistics regression*

Formatted Table

Variables	Categories	ICU admission Case (n=33) %	General admission Control (n=66) %	β	p value	Crude odds ratio	Adjusted odds ratio	95% CI for OR	
								Lower	Upper
Smoking	Non-smoker **	15 (23.4)	49 (76.6)		Ref.				
	Smoker	18 (51.4)	17 (48.6)	0.470	0.20	3.4	1.60	0.77	3.28
Blood pressure	Normal **	14 (20.6)	54 (79.4)		Ref.				
	Elevated	19 (61.3)	12 (38.7)	0.799	**0.03**	6.10	2.22	1.05	4.71
C reactive protein	Normal **	8 (14.8)	46 (85.2)		Ref.				
	Abnormal	25 (55.6)	20 (44.4)	1.073	**0.03**	7.18	2.92	1.24	6.84
Vaccination	Vaccinated **	14 (20.3)	55 (79.7)		Ref.				
	Un-vaccinated	19 (63.3)	11 (36.7)	0.633	0.11	6.78	1.88	0.86	4.10

Preventive practice	Good **	6 (18.2%)	49 (74.2%)		Ref.				
	Poor	27 (81.8%)	17 (25.8%)	1.206	**0.02**	12.97	3.34	1.19	9.31

*~~**: Reference, Cat: categories~~*

Formatted: Font: Not Bold, Italic

Formatted: Normal

Discussion

~~ICU admission rate among COVID-19 infected patients was found to be 31.13%~~ The proportion of ICU admission among COVID-19 infected patients was found to be 31.13%. I~~i~~n this study which was similar to the previous studies carried in Wuhan where 32% of COVID-19 infected patients were admitted to ICU because they required higher level of oxygen (2). Among the data collected from 20 regions the average ICU admission rate was 21.4%, range 9.4% - 45.9% (13). Another study, highlights that 20% of hospitalized patients with COVID-19 experiencing severe symptoms that required intensive care (14). Furthermore, a meta-analysis reported pooled rate of ICU admission was 32% in COVID -19 patients (15). Slight lower proportion of ICU admission was observed in a study done at South Korea, which reported that in total COVID-19 infected 9.5% of patients required ICU care and 4.6% patients actually admitted to ICU (16).

This study did not find any association between co-morbidities like Diabetes mellitus, hypertension, cardiac disease, COPD with ICU admission. Our findings were consistent with a recent studies done in China and United states, that did not found association between co-morbidities with ICU admission (2,17). A consistent findings was found in another study conducted in Spain, in which COPD and Asthma were not associated with ICU admission (3). Another cross- sectional analytical study conducted in tertiary care center of Nepal also found that co-morbidities were not associated with outcome of COVID-19 (18). In contrast to our findings, a study conducted in Milan, Italy and China found that co-morbidities, most commonly hypertension, cardiovascular disease, diabetes, COPD were related to independent risk factors for death in patient with COVID-19 (19,20). A similar study conducted in rural community hospitals eastern Virginia also revealed that diabetes mellitus and hypertension on admission were predictors of clinical deterioration leading to ICU admission (21). A meta-analysis done by Jianhua

Hu and Yanggan Wang also highlighted that prevalence of co-morbidities such as hypertension, CVD and diabetes mellitus in the severe COVID-19 patients was significantly higher than non-severe patients (22). These pathologies have been associated with greater probability of presenting complications and severe form of disease. The reported inconsistent and conflicting findings might be due to variable COVID-19 severity and mortality based on medical conditions specific to geographical regions. Supporting this statement, a systematic review and meta-analysis revealed that highest mortality among all co-morbidities was observed in European studies compared to Asian studies despite of lower prevalence of comorbidities (23). In addition, considerable variation in the prevalence of comorbidities and associated severity of disease in different geographical location was observed (23). Other potential reasons, for having not having significant association between co-morbidities with ICU admission are differences in the time of study and variation in the timing of COVID-19 spread. Majority of study were conducted during an early hit of pandemic especially in the elderly population and differences in variant and sub variant of coronavirus might have also created surge in findings. Sample size of the study, prevalence of comorbidities among COVID-19 patients, COVID-19 vaccination, diagnostic testing, reporting accuracy of co-morbidity might have also reported to inconsistent findings. Also the median age of case and control in this study was 59 years with compared to result of one meta-analysis where median age was over 65 years (22). This differences in age might also have role associated with co-morbidity and ICU admission.

Many previous studies found that COVID-19 patients present with lower oxygen saturation, high temperature, pulse rate, respiration rate and blood pressure at the time of hospital admission (17,18,20). These findings also correspond to the present study. Oxygen saturation is the indicator

or respiratory functions and demonstrate variations in arterial oxygenation to indicate hypoxemia in patients. A retrospective study showed that decreased oxygen saturation was significantly associated with ICU admission (17). This was again supported by a nested case- control study conducted in China, patient with $SpO_2 < 95.5\%$ have 2.35 odds of severe COVID-19 (14). Additionally, study conducted to find out risk factors for clinical deterioration in patients showed that decrease oxygen saturation at emergency department was the independent variable associated with clinical deterioration (3). A cross sectional comparative study conducted in survivors and non- survivors of Nepal also found that non- survivors tended to have lower oxygen saturation at admission (18).

This study also highlights elevated blood pressure at the time of hospital admission was independent risk factor in multivariable analysis. Patients with elevated blood pressure at admission were ~~9.88~~ 2.22 times more likely to be admitted to ICU than normal blood pressure. This finding was consistent with several studies done in China and Denmark where high systolic blood pressure were found to be significantly associated with treatment in ICU (2,24). Furthermore, a study conducted in Colombia to identify the factors associated with admission to ICU also found increased mean arterial pressure on admission significantly associated with patient infected with SARS – CoV- 2 (25).

Formatted: Strikethrough

Pulse rate was found to be significantly associated with ICU admission this finding was consistent with the study done in China where heart rate beats per minute was significantly associated with ICU admission (17). In bivariate analysis, this study found that febrile patients at the time of hospital admission were associated with admission to ICU. Findings was consistent with study done in China in which highest temperature of patients infected with COVID-19 was significant with requirement of ICU care (2). Additionally, Initial body temperature on admission were found

significant predictors to patient requiring intensive unit care (16). In this study, respiration rate was also found significantly associated with admission to ICU. This study showed that patient with tachypnea during the time of hospital admission were 15.5 times more likely to get admitted in ICU. Findings was alike with several studies where respiration rate was found significantly associated with admission to ICU (3,14,17).

Higher leucocyte count indicates our body is fighting infection or inflammation. In this study, total leucocyte count at the time of hospital admission was found to be associated with admission to ICU. Findings are consistent with study conducted in Italy where white blood cells count was significantly associated with risk of death (19). Similar findings was observed from multicenter retrospective case- control study, where WBC was significantly associated with non – survivor group (20). Furthermore, clinical mortality review done in Sukraraj Tropical and Infectious Disease Hospital, among 50 mortality reviewed 80% of patients had lymphocytopenia (26). A contrast finding was concluded by a study done in China where white blood cells were not significantly associated with severity of COVID-19 (14).

C- reactive protein is protein made by liver which is the sign of acute inflammation. In present study CRP was found as significant predictors of ICU admission. Findings was similar with study conducted in China where CRP was found as independent predictors associated with severe COVID-19 (14). Another study done in China also found CRP significantly associated with ICU admission (17). Recent meta-analysis also revealed that most common blood test abnormalities were elevated C- reactive protein(22).

COVID-19 vaccination with either first or second dose with any of available vaccine were not found associated with ICU admission in multivariate analysis. This findings was consistent with study done in central hospital of Nepal showed there was no significant association between

vaccination status with outcome of COVID-19 (18). In contrast to this findings, a study done in England reported vaccination was associated with a significant reduction in symptomatic SARS – CoV2 positive cases (27). The discrepancy in findings might be due to differences in natural immunity between people of different countries.

Present study concluded that patient admitted to ICU had longer hospital stay as compared to patient admitted in COVID-19 isolation ward, which is significantly associated. In this study, median hospital stay days among cases was 13 days and 2 days among control. This findings was consistent with the study conducted in Wuhan where the median time from onset of symptoms to ICU admission was 10.5 days (2). One more study conducted in China showed total in hospital days was 20 days among survivor and 15 days among non – survivor (20). Additionally another study conducted in Nepal, showed median ICU stay days was 10 days in survivors group and 8 days in non – survivor group (18). The possible explanation may be longer duration of stay in ICU due to disease severity, which resulted longer phase of recovery.

Present study found that application of preventive measures was found to be independently associated with ICU admission. Similar findings had been observed in systematic review and meta-analysis where non pharmaceutical Public health interventions showed 16.5% decrease in ICU admission (28). Another study conducted in Thailand also showed wearing masks, handwashing and social distancing were independently associated with lower risk for SARS – CoV-2 infection (29). A systematic review and meta- analysis revealed that several personal protective and social measures including physical distancing, hand washing and wearing masks were associated with reductions in the incidence of COVID -19 (30,31).

This study is of retrospective nature, participants may fail to remember the preventive measures they applied before two weeks of being infected with COVID -19. Thus, it may result in recall

Formatted: Left

bias. The present study was carried out with small sample size based on a single institution; ~~result may be generalized only to patient under similar setting~~ So, the results of the study can only be generalized to patients within the Sukraraj Tropical and Infectious Disease Hospital.

.

Formatted: Strikethrough

Conclusion

This study concludes that high blood pressure, abnormal C reactive protein~~,~~ and poor preventive practice ~~and longer duration of hospital stay~~ were the predictors associated with ICU admission. This study also identified the risk clinical features and lab investigation during the time of hospital admission. Evaluation of these factors during admission might help for risk assessment and early triage of potential severe COVID-19 cases so that best possible efforts can be made for prompt management of COVID – 19 cases.

Formatted: Strikethrough

Acknowledgement

We would like to acknowledge all participants who allocated time to our study and all people who directly and indirectly supported our study.

References

1. WHO Director-General's opening remarks at the media briefing on COVID-19 - 11 March 2020 [Internet]. [cited 2022 Mar 2]. Available from: https://www.who.int/director-general/speeches/detail/who-director-general-s-opening-remarks-at-the-media-briefing-on-covid-19---11-march-2020
2. Huang C, Wang Y, Li X, Ren L, Zhao J, Hu Y, et al. Clinical features of patients infected with 2019 novel coronavirus in Wuhan, China. Lancet. 2020;395(10223):497–506.
3. Uranga A, Villanueva A, Lafuente I, González N, Legarreta MJ, Aguirre U, et al. Risk factors for clinical deterioration in patients admitted for COVID-19: A case-control study. Rev Clínica Española (English Ed. 2022;222(1):22–30.
4. Hu Y, Liang W, Liu L, Li L. (No Title). n engl j med. 2020;18:1708–28.
5. Ministry of Health and Population. SitRep Health Sector Response to Novel Coronavirus (2019-nCoV) Confirm Result. 2020;(January):2020.
6. Ministry of Health and Population. SitRep. 2020.
7. Adhikari K, Gautam R, Pokharel A, Dhimal M. Insight into Delta variant dominated second wave of COVID-19 in Nepal. 2020;(January).
8. Ministry of Health and Population. Guidelines for Geriatric Population [Internet]. 2020. Available from: https://drive.google.com/file/d/1K7qNhAkzB2CGvFSIKe8WusjN_edHgCky/view
9. Lancet T. India under COVID-19 lockdown. Lancet [Internet]. 2020 [cited 2022 Mar 2];395:1315. Available from: https://www.
10. WHO. Clinical management Clinical management Living guidance COVID-19. 2021B. 2021;(January):16–44.
11. Interim Clinical Gudance for care of patients with COVID 19 in Healthcare Settings_05-04-2020.pdf.
12. Agarwal A, Ranjan P, Rohilla P, Saikaustubh Y, Sahu A, Dwivedi SN, et al. Development and validation of a questionnaire to assess preventive practices against COVID-19 pandemic in the general population. Prev Med Reports [Internet]. 2021;22(December 2020):101339. Available from: https://doi.org/10.1016/j.pmedr.2021.101339
13. Immovilli P, Morelli N, Antonucci E, Radaelli G, Barbera M, Guidetti D. COVID-19 mortality and ICU admission: The Italian experience. Crit Care. 2020;24:9–11.
14. Li X, Marmar T, Xu Q, Tu J, Yin Y, Tao Q, et al. Predictive indicators of severe COVID-19 independent of comorbidities and advanced age: A nested case -control study. Epidemiol Infect. 2020;
15. Mekonnen S, Id A, Ali SA, Mantfardo B, Basu B. Rate of Intensive Care Unit admission and outcomes among patients with coronavirus: A systematic review and Meta-analysis. 2020; Available from: https://doi.org/10.1371/journal.pone.0235653

16. Heo JN, Han D, Kim HJ, Kim D, Lee YK, Lim D, et al. Prediction of patients requiring intensive care for COVID-19: development and validation of an integer-based score using data from Centers for Disease Control and Prevention of South Korea. J Intensive Care. 2021;9(1):1–9.

17. Zhao Z, Chen A, Hou W, Graham JM, Li H, Richman PS, et al. Prediction model and risk scores of ICU admission and mortality in COVID-19. PLoS One [Internet]. 2020;15(7 July):1–14. Available from: http://dx.doi.org/10.1371/journal.pone.0236618

18. Simkhada N, Pathak BD, Bhusal L, Tandon OP, Shrestha A, Devkota S, et al. Comparison of clinical characteristics and short-term outcomes among COVID-19 patients in a tertiary care center during second wave pandemic in Nepal: a cross-sectional study. F1000Research. 2022;11:903.

19. Ciceri F, Castagna A, Rovere-Querini P, De Cobelli F, Ruggeri A, Galli L, et al. Early predictors of clinical outcomes of COVID-19 outbreak in Milan, Italy. Clin Immunol [Internet]. 2020;217(June):108509. Available from: https://doi.org/10.1016/j.clim.2020.108509

20. Gao J, Zhong L, Wu M, Ji J, Liu Z, Wang C, et al. Risk factors for mortality in critically ill patients with COVID-19: a multicenter retrospective case-control study. BMC Infect Dis. 2021;21(1):1–8.

21. Pathak V, Conklin C. Predictors of ICU Admission and Mortality in Patients with Coronavirus Disease - 2019 (COVID 19) in Community Hospitals. 2021;6:A2551–A2551.

22. Hu J, Wang Y. The Clinical Characteristics and Risk Factors of Severe COVID-19. Gerontology. 2021;67(3):255–66.

23. Thakur B, Dubey P, Benitez J, Torres JP, Reddy S. OPEN A systematic review and meta ‑ analysis of geographic differences in comorbidities and associated severity and mortality among individuals with COVID ‑ 19. Sci Rep [Internet]. 2021;1–13. Available from: https://doi.org/10.1038/s41598-021-88130-w

24. Israelsen SB, Kristiansen KT, Hindsberger B, Ulrik CS, Andersen O, Jensen M. Characteristics of patients with COVID-19 pneumonia at Hvidovre Hospital, March- April 2020. Denish Med J. 2020;(April):28–31.

25. Enrique Machado-Alba JI, Fernando Valladales-Restrepo L, Enrique Machado-Duque M, Gaviria-Mendoza A, Sá nchez-Ramírez N, Felipe Usma-Valencia A, et al. Factors associated with admission to the intensive care unit and mortality in patients with COVID-19, Colombia. 2021; Available from: https://doi.org/10.1371/journal.pone.0260169

26. Bastola, A.; Shrestha, S.; Nepal, R.; Maharjan, K.; Shrestha, B.; Chalise, B.S.; Thapa, P.; Balla, P.; Sapkota A. S. Clinical Mortality Review of COVID-19 Patients at Sukraraj Tropical and Infectious Disease Hospital, Nepal; A retrospective Study. Trop Med Infect Dis. 2021;

27. Bernal JL, Andrews N, Gower C, Stowe J, Robertson C, Tessier E, et al. Early effectiveness of COVID-19 vaccination with BNT162b2 mRNA vaccine and ChAdOx1 adenovirus vector vaccine on symptomatic disease, hospitalisations and mortality in older

adults in England. medRxiv [Internet]. 2021;2021.03.01.21252652. Available from: https://doi.org/10.1101/2021.03.01.21252652

28. 1☯ SI, Gholipour K, 2☯ ID, Azami-Aghdash S, Ghiasi A, Rezapour A, et al. Effectiveness of non-pharmaceutical public health interventions against COVID-19: A systematic review and meta-analysis. 2021;

29. Doung-Ngern P, Suphanchaimat R, Panjangampatthana A, Janekrongtham C, Ruampoom D, Daochaeng N, et al. Case-Control Study of Use of Personal Protective Measures and Risk for SARS-CoV 2 Infection, Thailand. Emerg Infect Dis. 2020;26(11):2607–16.

30. Talic S, Shah S, Wild H, Gasevic D, Maharaj A, Ademi Z, et al. Effectiveness of public health measures in reducing the incidence of covid-19, SARS-CoV-2 transmission, and covid-19 mortality: Systematic review and meta-analysis. BMJ. 2021;375:1–15.

31. Regmi K, Lwin CM. Factors associated with the implementation of non-pharmaceutical interventions for reducing coronavirus disease 2019 (Covid-19): A systematic review. Int J Environ Res Public Health. 2021;18(8):1–27.

List of legend

Table 1: Association between sociodemographic characteristics with ICU admission

Table 2: Association between Co-morbidities and behavioral characteristics with ICU admission

Table 3: Severity of COVID-19 at the time of hospital admission

Table 4: *Association between clinical and lab parameter with ICU admission*

Table 5: Association between preventive practice and ICU admission

Table 6: *Conditional logistics regression*

Formatted: Font: Bold

Formatted: Font: Times New Roman, 12 pt, Bold, Italic

Formatted: Font: Calibri, 11 pt, Not Bold, Italic, Font color: Auto

Formatted: Normal, Line spacing: single

Formatted: Font: Calibri, 11 pt, Font color: Auto

Formatted: Font: Bold

Formatted: Normal, Left, Line spacing: single

Formatted: Font: Calibri, 11 pt, Font color: Auto

Annexure - I

Questionnaire to assess prevention practices against COVID-19 in the general population

Kindly fill the questionnaire below based on your routine for last two weeks.

SN	Item	Options				
1	How often do you shake hands while greeting people nowadays?	Always (more than 90% times)	Mostly (Approx 75% times)	Commonly (Approx 50% times)	Occasionally (approx. 25% times)	Rarely (less than 10% times)
2	How often do you wash/sanitize your hands with soap and water/alcohol-based sanitizer?	Always (more than 90% times)	Mostly (Approx 75% times)	Commonly (Approx 50% times)	Occasionally (approx. 25% times)	Rarely (less than 10% times)
3	How often do you ensure that you wash/sanitize your hands for at least 20s?	Always (more than 90% times)	Mostly (Approx 75% times)	Commonly (Approx 50% times)	Occasionally (approx. 25% times)	Rarely (less than 10% times)
4	How often do you ensure that you cover your face with a handkerchief/bent elbow while coughing/sneezing?	Always (more than 90% times)	Mostly (Approx 75% times)	Commonly (Approx 50% times)	Occasionally (approx. 25% times)	Rarely (less than 10% times)
5	How often do you ensure that you clean your hands before touching your eyes/nose/mouth?	Always (more than 90% times)	Mostly (Approx 75% times)	Commonly (Approx 50% times)	Occasionally (approx. 25% times)	Rarely (less than 10% times)
6	How often do you maintain a minimum distance of one meter at your workplace?	Always (more than 90% times)	Mostly (Approx 75% times)	Commonly (Approx 50% times)	Occasionally (approx. 25% times)	Rarely (less than 10% times)

7	How often do you maintain a minimum distance of one meter while eating food with your colleagues at your workplace?	Always (more than 90% times)	Mostly (Approx 75% times)	Commonly (Approx 50% times)	Occasionally (approx. 25% times)	Rarely (less than 10% times)
8	How often do you avoid going out of the house unnecessarily?	Always (more than 90% times)	Mostly (Approx 75% times)	Commonly (Approx 50% times)	Occasionally (approx. 25% times)	Rarely (less than 10% times)
9	How often have you attended social gathering in the past two months? (Like meeting friends, going to religious places, visiting malls, theaters etc)	Never (5)	Once (4)	Twice (3)	Thrice (2)	More than three times (1)
10	How often do you maintain distance of one meter in public spaces (eg grocery shopping, social gathering etc) ?	Always (more than 90% times)	Mostly (Approx 75% times)	Commonly (Approx 50% times)	Occasionally (approx. 25% times)	Rarely (less than 10% times)
11	How often do you wear masks while going out of home?	Always (more than 90% times)	Mostly (Approx 75% times)	Commonly (Approx 50% times)	Occasionally (approx. 25% times)	Rarely (less than 10% times)
12	While wearing a mask, how often do you ensure that both your nose and mouth are covered?	Always (more than 90% times)	Mostly (Approx 75% times)	Commonly (Approx 50% times)	Occasionally (approx. 25% times)	Rarely (less than 10% times)
13	How often do you keep your mask properly in a separate bag/dustbin after using it?	Always (more than 90% times)	Mostly (Approx 75% times)	Commonly (Approx 50% times)	Occasionally (approx. 25% times)	Rarely (less than 10% times)
14	How often do you sanitize your personal items (eg purse/mobile phone, etc) with sanitizer when you come home?	Always (more than 90% times)	Mostly (Approx 75% times)	Commonly (Approx 50% times)	Occasionally (approx. 25% times)	Rarely (less than 10% times)
15	How often do you take precautions when buying things to avoid virus contamination?	Always (more than 90% times)	Mostly (Approx 75% times)	Commonly (Approx 50% times)	Occasionally (approx. 25% times)	Rarely (less than 10% times)

16	How often do you obey government restrictions regarding the COVID-19 pandemic?	Always (more than 90% times)	Mostly (Approx 75% times)	Commonly (Approx 50% times)	Occasionally (approx. 25% times)	Rarely (less than 10% times)
17	In case you develop symptoms of the disease, you will contact the hospital/helpline/authority regarding it?	Strongly agree	Agree	Can’t say	Disagree	Strongly disagree
18	If you come in contact with COVID positive/suspect person, you would stop going to work and confine yourself to the home away from friends and family members.	Strongly agree	Agree	Can’t say	Disagree	Strongly disagree

Annexure – II

Nepali version of Preventive practice questionnaire

1. आजकल तपाई मानिसहरूलाई अभिवादन गर्दा कति पटक हात मिलाउनुहुन्छ?
    - सधैं)९०% भन्दा बढी पटक(
    - प्रायः)लगभग ७५% पटक(
    - सामान्यतया)लगभग ५०% पटक(
    - कहिलेकाहीँ)लगभग २५% पटक(
    - विरलै)१०% पटक भन्दा कम(

2. तपाइँ कति पटक साबुन र पानी/अल्कोहलमा आधारित सेनिटाइजरले तपाइँको हात धुनुहुन्छ / सेनिटाइज गर्नुहुन्छ ?
    - सधैं)९०% भन्दा बढी पटक(
    - प्रायः)लगभग ७५% पटक(
    - सामान्यतया)लगभग ५०% पटक(
    - कहिलेकाहीँ)लगभग २५% पटक(
    - विरलै)१०% पटक भन्दा कम(

3. तपाईले कम्तिमा 20 सेकेन्डसम्म आफ्नो हात धुने/सनिटाइज गर्ने कुरा कति पटक सुनिश्चित गर्नुहुन्छ?
    - सधैं)९०% भन्दा बढी पटक(
    - प्रायः)लगभग ७५% पटक(
    - सामान्यतया)लगभग ५०% पटक(
    - कहिलेकाहीँ)लगभग २५% पटक(

- विरलै)१०% पटक भन्दा कम(

4. तपाईले खोक्दा/हाछ्युँ गर्दा कति पटक यो सुनिश्चित गर्नुहुन्छ कि तपाईले आफ्नो अनुहार रुमाल/ कुहिनोले छोप्नु हुन्छ ?
    - सधैं)९०% भन्दा बढी पटक(
    - प्रायः)लगभग ७५% पटक(
    - सामान्यतया)लगभग ५०% पटक(
    - कहिलेकाहीँ)लगभग २५% पटक(
    - विरलै)१०% पटक भन्दा कम(

5. तपाइँ कति पटक तपाइँको आँखा / नाक / मुख छुनु अघि तपाइँको हातहरू सफा छ/छैन भनेर सुनिश्चित गर्नुहुन्छ ?
    - सधैं)९०% भन्दा बढी पटक(
    - प्रायः)लगभग ७५% पटक(
    - सामान्यतया)लगभग ५०% पटक(
    - कहिलेकाहीँ)लगभग २५% पटक(
    - विरलै)१०% पटक भन्दा कम(

6. तपाईं आफ्नो कार्यस्थलमा न्यूनतम एक मिटरको दुरी कति पटक कायम गर्नुहुन्छ?
    - सधैं)९०% भन्दा बढी पटक(
    - प्रायः)लगभग ७५% पटक(
    - सामान्यतया)लगभग ५०% पटक(
    - कहिलेकाहीँ)लगभग २५% पटक(
    - विरलै)१०% पटक भन्दा कम(

7. तपाईको कार्यस्थलमा आफ्ना सहकर्मीहरूसंग खाना खाँदा कति पटक न्यूनतम एक मिटरको दुरी कायम राख्नुहुन्छ ?
    - सधैं)९०% भन्दा बढी पटक(
    - प्रायः)लगभग ७५% पटक(
    - सामान्यतया)लगभग ५०% पटक(
    - कहिलेकाहीँ)लगभग २५% पटक(
    - विरलै)१०% पटक भन्दा कम(

8. अनावश्यक रूपमा घरबाहिर ननिस्कन कत्तिको बेवास्ता गर्नुहुन्छ ?

- सधैं)९०% भन्दा बढी पटक(
- प्रायः)लगभग ७५% पटक(
- सामान्यतया)लगभग ५०% पटक(
- कहिलेकाहीँ)लगभग २५% पटक(
- विरलै)१०% पटक भन्दा कम(

9. विगत दुई महिनामा तपाईले कति पटक सामाजिक जमघटमा भाग लिनुभयो? (जस्तै बैठक, साथीहरू भेट्ने , धार्मिक स्थलहरूमा जाने, मल, थिएटरहरू, आदि)

- कहिले पनि छैन
- एक चोटी
- दुई चोटी
- तिन चोटी
- तिन भन्दा धेरै

10. तपाईले कति पटक सार्वजनिक स्थानहरूमा न्यूनतम एक मिटरको दूरी कायम गर्नुहुन्छ)जस्तै। किराना किनमेल, सामाजिक जमघट, आदि(?
    - सधैं)९०% भन्दा बढी पटक(
    - प्रायः)लगभग ७५% पटक(
    - सामान्यतया)लगभग ५०% पटक(
    - कहिलेकाहीँ)लगभग २५% पटक(
    - विरलै)१०% पटक भन्दा कम(

11. घरबाहिर निस्कँदा कति पटक मास्क लगाउनुहुन्छ ?
    - सधैं)९०% भन्दा बढी पटक(
    - प्रायः)लगभग ७५% पटक(
    - सामान्यतया)लगभग ५०% पटक(
    - कहिलेकाहीँ)लगभग २५% पटक(
    - विरलै)१०% पटक भन्दा कम(

12. मास्क लगाउँदा, तपाइँ कति पटक यो सुनिश्चित गर्नुहुन्छ कि तपाइँको नाक र मुख दुबै छ ढाकिएको छ ?
    - सधैं)९०% भन्दा बढी पटक(
    - प्रायः)लगभग ७५% पटक(

- सामान्यतया)लगभग ५०% पटक(
- कहिलेकाहीँ)लगभग २५% पटक(
- विरलै)१०% पटक भन्दा कम(

13. तपाईंले कति पटक आफ्नो मास्क प्रयोग गरिसकेपछि अलग्गै झोला/डस्टबिनमा राम्ररी राख्नुहुन्छ?

- सधैं)९० % भन्दा बढी पटक(
- प्रायः)लगभग ७५% पटक(
- सामान्यतया)लगभग ५०% पटक(
- कहिलेकाहीँ)लगभग २५% पटक(
- विरलै)१०% पटक भन्दा कम(

14. तपाई घर आइसके पछि आफ्नो व्यक्तिगत वस्तुहरू)जस्तै पर्स/मोबाइल फोन, इत्यादि(कति पटक सेनिटाइजरले सेनिटाइज गर्नुहुन्छ ?

- सधैं)९०% भन्दा बढी पटक(
- प्रायः)लगभग ७५% पटक(
- सामान्यतया)लगभग ५०% पटक(
- कहिलेकाहीँ)लगभग २५% पटक(
- विरलै)१०% पटक भन्दा कम(

15. सामान किन्दा भाइरस संक्रमणबाट बच्न कति पटक सावधानी अपनाउनुहुन्छ?

- सधैं)९०% भन्दा बढी पटक(
- प्रायः)लगभग ७५% पटक(
- सामान्यतया)लगभग ५०% पटक(
- कहिलेकाहीँ)लगभग २५% पटक(
- विरलै)१०% पटक भन्दा कम(

16. कोभिड-१९ महामारीको सन्दर्भमा सरकारी प्रतिबन्धहरूको कत्तिको पालना गर्नुहुन्छ?

- सधैं)९०% भन्दा बढी पटक(
- प्रायः)लगभग ७५% पटक(
- सामान्यतया)लगभग ५०% पटक(
- कहिलेकाहीँ)लगभग २५% पटक(
- विरलै)१०% पटक भन्दा कम(

17. यदि तपाईंलाई रोगको लक्षणहरू देखा परेमा, तपाईंले यस सम्बन्धमा अस्पताल/हेल्पलाइन/ अख्तियार मा सम्पर्क गर्नुहुनेछ ?

- एकदमै सहमत

- सहमत
- भन्न सक्दिन
- असहमत
- एकदमै असहमत

18. दि तपाइँ कोभिड पोजिटिभ/शङ्कास्पद व्यक्तिको सम्पर्कमा आउनुभयो भने, तपाइँ काममा जान बन्द गरि आफूलाई साथीहरू र परिवारका सदस्यहरूबाट टाढा घरमा सीमित राख्नु हुन्छ ?
    - एकदमै सहमत
    - सहमत
    - भन्न सक्दिन
    - असहमत
    - एकदमै असहमत
        - अन्य कारणहरू)कृपया निर्दिष्ट गर्नुहोस्(:

Date: 18/5/023

To,
The Editor
PLOS Global Public Health

Thank you so much for reviewing my manuscript thoroughly. I wanted to give justification about resubmission about previously rejected manuscript. The major reason for rejection was the issue with the date of ethical clearance.

Respected Sir/ Ma'am, at first, I want to let you know that this study is my thesis work of Master's in Public health. In our thesis, we count the study period from proposal writing phase to report preparation. I have mentioned that study starts from April doesn't mean data was collected from April. Proposal preparation was started from April but the actual data collection was done after the ethical clearance only. To assure you about my statement, I have also attached a letter from my study site which is the only central Tropical and Infectious disease hospital in Nepal, they do not allow for data collection unless the ethical clearance.

Regarding your next query of informed consent, as in my study data collection for preventive practice was collected through telephone interview for this purpose due to the retrospective nature of study verbal consent was taken. After getting verbal consent from each patient, collection of clinical and preventive predictors was done.

I also want to let you know that both the institute where I study and the hospital where I conducted my study are renowned health institutions in Nepal, they never let anyone conduct study without ethical clearance.

If you want to further verify you can directly contact both institutions BP Koirala Institute of health sciences and Sukraraj Tropical and Infectious Disease hospital in Nepal.

I apologize for my mistake; I am a student and this is my first attempt to publish a paper. I write the study period honestly from the student perspective, as we count the study period from the proposal writing phase.
I hope you have understood and consider it as a beginner's unintentional mistake. I would assure you that this is the greatest learning for beginners like me and I wouldn't repeat such mistakes in my whole career.
I hope you have understood.

I have revised and clearly mentioned the date issue in the resubmitted manuscript along with supporting letter from my study site.
I have mentioned all the genuine and true justification from my side, but the final decision will be yours.

Thank you

Regards
Dipsikha Aryal
Nepal

Dear Editor and Reviewers,

Thank you so much for this wonderful consideration and we highly appreciate your meticulous review and constructive comments and suggestion. We have revised our manuscript incorporating your comments and suggestions. Here we write a rebuttal letter that responds to each point raised by the Editor and Reviewers. Please kindly consider our responses.

**Comments**	**Response**
**Journal requirements**	
1. In the ethics statement in the Methods, you have specified that verbal consent was obtained. Please provide additional details regarding how this consent was documented and witnessed, and state whether this was approved by the IRB.	The verbal consent was taken and documented by the following ways:<br><br>At first researcher collected list of participants meeting the inclusion criteria with contact details. We have directly contacted with each respondent, at first introduction of researcher and the main aim of the study were clearly explained, interview will last for around 10 – 15 minutes were clarified. Asked respondents about their most favorable time and contacted accordingly.<br><br>Participants information sheet was prepared during the time of proposal development, all the information to respondents about voluntary participation and confidentiality were provided in Nepali language. The way of taking consent were consistent to all the respondents. The date and time of consent provided by the respondents is recorded by researcher. This is

	approved by Institutional ethical review committee of BPKIHS (Reference number 236/078/079-IRC).<br><br>The main reason for verbal consent with the retrospective nature of study.<br><br>If required for further confirmation and assurance proposal submitted to IRC and participants information sheet can be uploaded.
2. Please provide separate figure files in .tif or .eps format only and remove any figures embedded in your manuscript file. Please also ensure all files are under our size limit of 10MB.	Yes. We have incorporated as indicated.
3. We have noticed that you have uploaded Supporting Information files, but you have not included a list of legends. Please add a full list of legends for your Supporting Information files after the references list.	Legends added for flow chart.
4. Please amend your Data Availability Statement and indicate where the data may be found.	Data can be found in .osf, link of data osf attached below.<br>https://osf.io/az4wr/
**Reviewer’s comment**	

Reviewer # 1	
1. Methods section needs restructuring. Would benefit from a list of inclusions and exclusions early on, to include age restrictions and outcome (in-hospital death). Sample selection: Line 193 From my understanding, the 99 patients were not randomly selected. Rather, they identified ICU admissions with COVID-19 (who survived discharge) and then matched them to other patients with admitted to the ward with COVID-19. Lines 109-110: I think they are saying every 2nd or every 3rd. It is probably every 3rd patient was reviewed.	Inclusion and exclusion criteria have been added in line 147 – 167 of revised manuscript.<br><br>Line 193<br><br>Response: Only 33, that is ICU admitted patients were randomly selected according systematic random sampling. Rest 66 control were selected as per age and sex matched with case.<br><br>Line 109 – 110<br><br>Response: Yes, every 3rd file was reviewed for ICU admitted case. Which is done via systematic random sampling method.
Lines 114-115. They mention "inclusion criteria", but I do not see where those are listed. I think they are trying to say they selected 2 controls based on each case. Analysis: Lines 242-245 belong in the methods section "variables that were significantly associated with ICU admission, with a p-value 243 less than or equal to 0.2 in bivariate analysis were taken for multivariate logistics regression 244 analysis. Variables with	Line 114-115<br><br>Response: Thank you for the suggestion. Inclusion and exclusion criteria have been added in line 147 – 167 of revised manuscript.<br><br>Lines 242-245<br><br>Response: Reanalysis of data has performed; conditional logistics regression has done as per suggestion instead of binary logistics regression.

expected count less than 5 were not included in multivariate logistics 245 regression analysis.”	Independent variables found statistically significant (p<0.05) in bivariate analysis were only further utilized in conditional logistics regression.<br><br>The “p-value less than or equal to 0.2 in bivariate analysis were taken for multivariate logistics” this statement has been removed in revised manuscript line number 280 – 283.
2. Lines 120-126 these are results (as per Figure 1).	Response: Yes, this is explanation of figure 1.
3. Figure 1- it is surprising that all deaths occurred in ward patients. They should check that.	Response: As mentioned on line 133 – 134 of revised manuscript, the number 105 deaths are the total death during the course of treatment in hospital. The arrow in flow chart gives the unclear information and it shows that death occurred in ward only, which in fact is not true. The arrow is restructure as per comments.<br><br>In conclusion, 105 is total death in hospital during six months including both ICU admitted and general admission.

Also, why are the ICU patients separated into the ventilator group? Were those cases ineligible for the study as well?	Response: Patient who required ventilator were also included in the study. The ventilator group were separated in flow chart just to make clear picture about the ICU admitted group.<br><br>Flowchart is restructured and uploaded as .tif file.
1. Recall bias – Data collection occurred in June – July 2022. The population contacted were in hospital between July 2021 and Jan 2022. This suggests telephone interviews asking about symptoms and preventative measures in the 2 weeks prior to their hospitalisation occurred between six months and a year after their hospitalisation. Preventative measures recall may be worse and/or biased for ICU patients compared to ward patients. Some variables are likely okay to collect six months after hospitalisation– e.g. smoking, comorbidities and vaccination status. I would not conclude anything, however, about other variables due to bias.	Response: The main limitation of the study with retrospective design is recall bias, in this study as well main limitation is recall bias. However, researcher has tried several techniques to minimize the recall bias, administration of structured questionnaire and interview to both case and control, consistency in the pattern of preventive practice questions, also the direct leading question was avoided.<br><br>Limitation of this study is mentioned in line 414 – 415 of revised manuscript.
2. Data analysis: The analysis they did was flawed, hence I cannot yet believe the findings. Logistic regression model likely needed to be conditional due to the matching procedure. Inclusion of the matching variables (age and sex) is required at a minimum for matched studies. How was inter-dependence of	Response: Thank you for the comment. This comment has been addressed, according to suggestion. Re analysis of data set has performed, conditional logistic regression has done instead of binary logistics regression. Results are presented in table 6 of revised manuscript.

variables assessed? Eg. Smoking and preventative practices? Smoking and sex? Vaccination status and smoking? With a sample of only 100 in total, no more than 5 or 6 covariates should be included. Sex and age need to be included, then they can try and build a parsimonious model with other variables with a large B-coefficient, after assessing for interdependence/ intercorrelations. Did they test COPD in multivariable model? And COPD was likely associated with smoking. Hence they need to include the interaction term of COPD*smoking or pick the better predictor of the two. The Nagelkerke R2 is very high. I would be surprised if the variables in the model explained that much of the variance. Furthermore, duration of hospital stay isn't a predictor of ICU admission, it's likely a result of ICU admission. That should not be included.	
They discuss "severity at admission" in the methods section but do not present that data anywhere in the tables. It would be interesting to see that data, especially if the ward patients were the ones that were dying. Did patients with a "do not resuscitate" plan go to the ward directly?	Response: Thank you for the comment, sir/ma'am. The disease severity at the time of hospital admission is added in table 3 with diagnosis (recorded from the hospital file) and description.

Age categories would also be helpful to see, e.g. 18-39 years, 40-59 years, 60-79 years and 80+ years.	Response: Age group according to category 18-39 years, 40-59 years, 60-79 years and 80+ years is added in table 1.
**Reviewer #2**	
Minimum data was not provided, therefore it is impossible to verify the results.	Thank you for the comment. Data is made available in data availability system.
Study site and population not properly described.	Detail description of study site in line 98 - 107 and population in line 147 – 167 has been added in methodology section of revised manuscript.
The flowchart contains errors, as the numbers do not add up.<br>The flowchart follows a cohort format, which is an inappropriate study design.<br>It is recommended to approach the work as a retrospective cohort design or a cross-sectional analysis instead of a case-control study.	Total COVID-19 patients during six months = 713<br>Excluding <18 years (713 – 15 = 698)<br>Among 698 (ICU admitted = 222, General admission = 371) (Remaining 105 total death during the course of treatment)<br>That is 222 + 371 + 105 = 698<br>Among (222 + 371 = 593), initially selected 141 patients from 593, within them 33 case and 66 control were enrolled in study remaining were excluded due to unmatched.<br><br>Flowchart is restructured and submitted as .tif file. Patient enrollment criteria is clearly written in descriptive form and flowchart is drawn to make clear picture. If flowchart makes information confusing it can be deleted.

What was the power of the study calculated? The sample size (N) appears too small.	Taking reference of case control study done in Spain on 2021 (ref 3),<br>proportion of cases (p1) is 33.33% and proportion of control (p2) is 66.67%.<br>Formula, $n= (Z_{\alpha/2} + Z_{\beta})^{2} (p_1q_1 + p_2q_2) / (p_1\text{-}p_2)^{2}$<br>$p_1$= 33.33% that is 0.33,<br>$q_1$ = 0.67 p2= 66.67% that is 0.66,<br>q_2= 0.34<br>$Z_{\alpha/2}$ = 1.96 at 95% Confidence Interval<br>Z_{β}= 0.84 for 80% power<br>Now using formula,<br>$n= (1.96 + 0.84)^2 (0.33 * 0.67 + 0.66 * 0.34) / (0.33\text{-}0.66)^2$<br>= 7.84 (0.2211 + 0.2244) /0.1089<br>= 3.492 / 0.1089<br>= 32.07 ~ 33<br>Therefore, the calculated sample size for cases was 33 and control was 66 (1:2 ratio). Total number of samples was 99.
The study sample size was small; consequently, the statistical results yielded extremely wide confidence intervals. While these intervals allow us to determine whether a variable is associated with the model, they do not facilitate additional interpretations that contribute to understanding the risk probability.	Thank you for the comment. Yes, we did re-analysis of data set. Conditional logistic regression is performed instead of binary logistics, with the proper statistical technique the confidence interval is not wide.<br><br>This is the final results with confidence interval:<br>Patients with elevated Blood pressure (AOR = 2.22; 95% CI 1.05 – 4.71, p 2.2= 0.015), Abnormal CRP (AOR = 2.92; 95% CI 1.24 –

	6.84, p = 0.012) at the time of hospital admission were more likely to get admitted to ICU. Patient with poor preventive practice (AOR = 3.34; 95% CI 1.19 – 9.31, p=0.02) more likely to get admitted to ICU than patient with good preventive practice.<br><br>This is incorporated in revised manuscript.
The abstract mentions "The adjusted multivariable logistics regression," but this information is not thoroughly explained in the methods section. Furthermore, the "multivariable logistics regression" only appears in Table 5, where it is referred to as 'binary logistic regression'. It is unclear whether the authors used binary or multiple logistic regression.	This comment has been addressed and incorporated in revised manuscript.<br><br>For bivariate analysis chi-square test and Mann- Whitney U test was performed.<br>Reanalysis of data has done and conditional logistic regression is performed in revised manuscript.
Report the number of instances of "missing data" for each variable.	Missing data were excluded prior to data analysis.
Include both crude and adjusted odds in the same Table 5.	We have incorporated COR and AOR in the same table, which is table number 6 in revised manuscript.
The reference levels for each variable in the logistic regression (Tables 3 & 4) are not clearly specified.	Thank you for the comment. Reference category has been added in each variable which is denoted by asterisk (*) symbol.<br>In the revised manuscript, table number 4 and 5.

For the variable "COVID-19 vaccination" (Yes/No), the reference level should be set as "Yes." This adjustment allows the "No" value to indicate the significantly increased risk for non-vaccinated individuals being hospitalized in the ICU.	Yes, the reference category is yes in "COVID-19 vaccination".<br><br>Change is incorporated in revised manuscript.
Line 371: The sentence "result may be generalized only to patient under similar setting" is incorrect. The study's results cannot be generalized to patients under similar settings. The results of the study can only be generalized to patients within the same hospital.	Corrected as per suggestion.
**Minor Changes**	
Table 4 is incorrectly numbered.	Corrected.
In Table 5, provide the number of cases and controls for each variable.	Number of cases and control added, which is in table 6 of revised manuscript.
Line 51: Change "pandemic in March 2020(1)" to "pandemic in March 2020 (1)."	Changed incorporated in revised manuscript in line 52.
Line 55: Change "major symptoms like fever, cough, dyspnea" to "major symptoms such as fever, cough, and dyspnea."	Changed incorporated in revised manuscript in line 55.
Line 56: Change "cutaneous manifestations are common clinical features of this disease (2)" to "cutaneous manifestations are common clinical features of this disease (2)."	Same
Line 64: Change "The first case of coronavirus was seen in a Nepalese Citizen returning" to	Changed incorporated in revised manuscript in line 64.

"The first case of coronavirus was observed in a Nepalese citizen returning."	
Line 69: Change "endorsed in Nepal from 25th March 2020 (6)" to "endorsed nationwide in Nepal on March 25, 2020 (6)."	Changed incorporated in revised manuscript in line 69.
Line 242: Change "variables that were significantly associated with ICU admission" to "variables significantly associated with ICU admission."	Changed incorporated in revised manuscript in line 290.
Line 104: Change "public hospital with High Dependency Unit" to "public hospital with a High Dependency Unit."	Changed incorporated in revised manuscript in line 116.
Line 259: Change "COVID-19 infected patients was found to be 31.13%" to "The proportion of COVID-19 infected patients was found to be 31.13%.	Changed incorporated in revised manuscript in line 302 – 303.

Thank you

Corresponding author

Data Review URL

Click here to download Data Review URL http://osf.io/az4wr/
