# Supplementary material for: Predictors to Intensive Care Unit admission among patient with coronavirus disease in Sukraraj Tropical and Infectious Disease Hospital, Nepal: A case-control study
Source: PLOS Glob Public Health. 2024 Mar 21;4(3):e0002516. doi: 10.1371/journal.pgph.0002516 (PMC10957074; doi:10.1371/journal.pgph.0002516)
Supplement: Attachment — Submitted filename: Flowchart and Map missing information.pdf [file pgph.0002516.s007.pdf]

Flowchart missing information

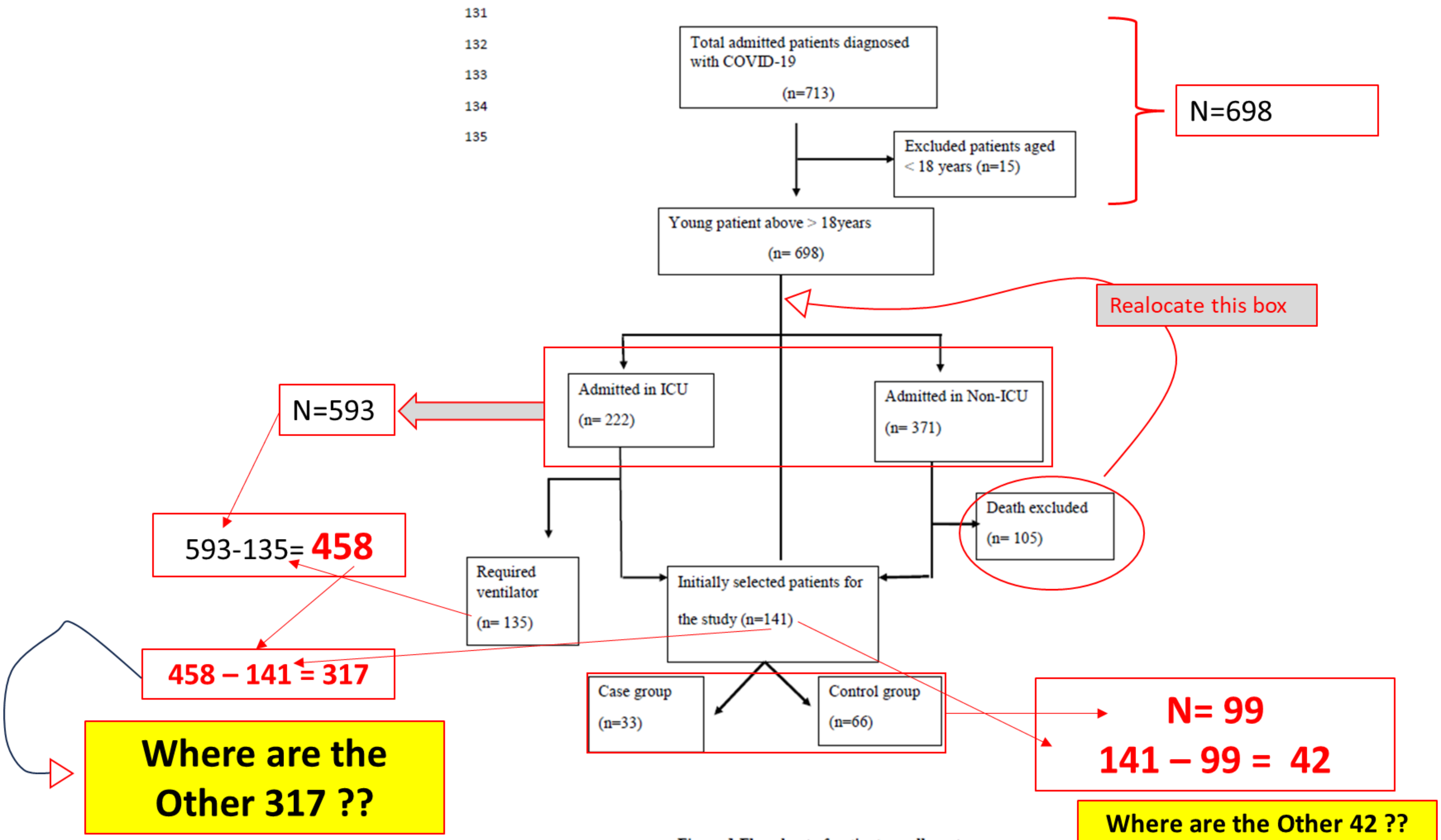

Figure 1 Flowchart of patient enrollment

Map’s missing information

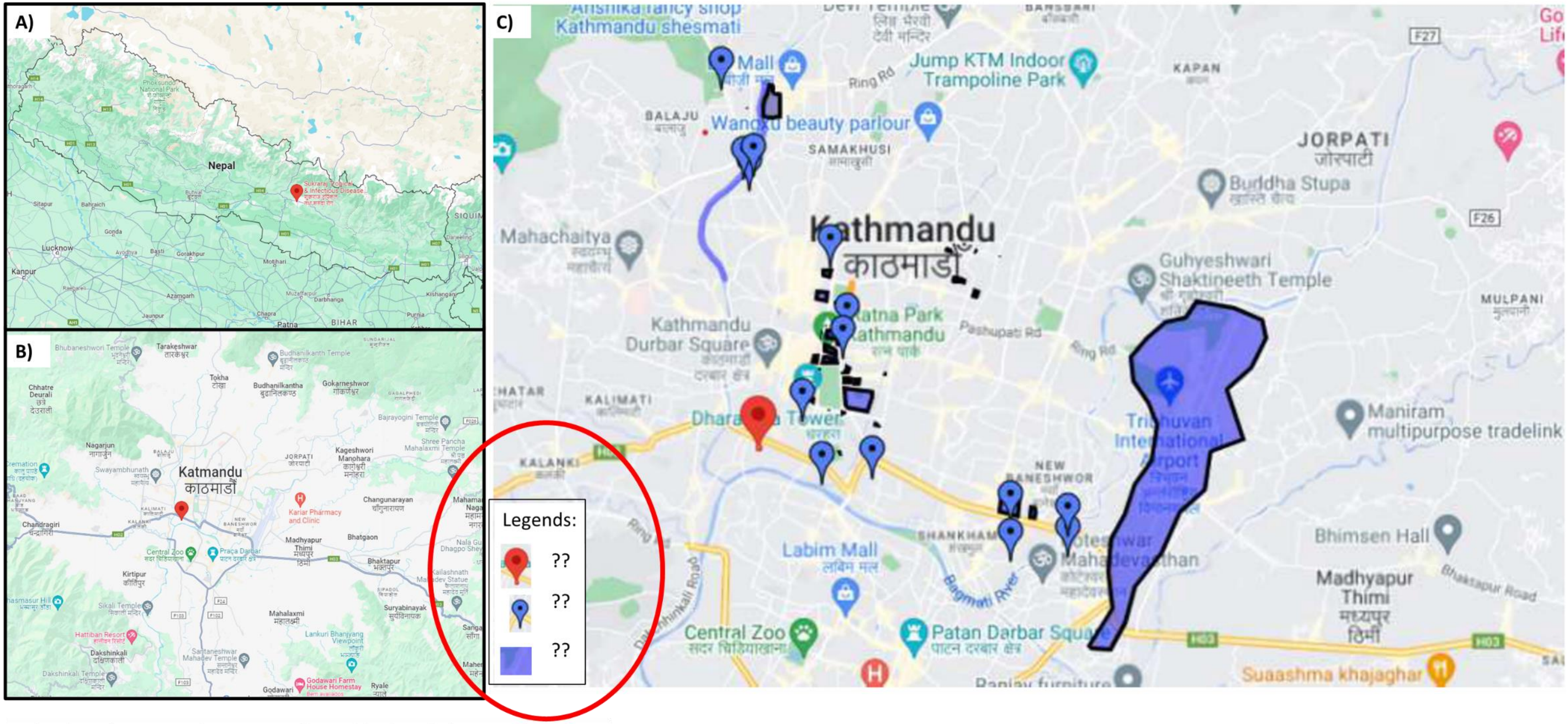

Subtitles: A) Countries' Locations B) Neighborhood C) Hospital Location
