# Supplementary material for: Predictors to Intensive Care Unit admission among patient with coronavirus disease in Sukraraj Tropical and Infectious Disease Hospital, Nepal: A case-control study
Source: PLOS Glob Public Health. 2024 Mar 21;4(3):e0002516. doi: 10.1371/journal.pgph.0002516 (PMC10957074; doi:10.1371/journal.pgph.0002516)
Supplement: Attachment — Submitted filename: Reason for resubmission.docx [file pgph.0002516.s003.docx]

Date: 18/5/023

To,

The Editor

PLOS Global Public Health

Thank you so much for reviewing my manuscript thoroughly. I wanted to give justification about resubmission about previously rejected manuscript. The major reason for rejection was the issue with the date of ethical clearance.

Respected Sir/ Ma'am, at first, I want to let you know that this study is my thesis work of Master's in Public health. In our thesis, we count the study period from proposal writing phase to report preparation. I have mentioned that study starts from April doesn't mean data was collected from April. Proposal preparation was started from April but the actual data collection was done after the ethical clearance only. To assure you about my statement, I have also attached a letter from my study site which is the only central Tropical and Infectious disease hospital in Nepal, they do not allow for data collection unless the ethical clearance.

Regarding your next query of informed consent, as in my study data collection for preventive practice was collected through telephone interview for this purpose due to the retrospective nature of study verbal consent was taken. After getting verbal consent from each patient, collection of clinical and preventive predictors was done.

I also want to let you know that both the institute where I study and the hospital where I conducted my study are renowned health institutions in Nepal, they never let anyone conduct study without ethical clearance.

 If you want to further verify you can directly contact both institutions BP Koirala Institute of health sciences and Sukraraj Tropical and Infectious Disease hospital in Nepal.

I apologize for my mistake; I am a student and this is my first attempt to publish a paper. I write the study period honestly from the student perspective, as we count the study period from the proposal writing phase.

I hope you have understood and consider it as a beginner's unintentional mistake.  I would assure you that this is the greatest learning for beginners like me and I wouldn't repeat such mistakes in my whole career.

I hope you have understood.

I have revised and clearly mentioned the date issue in the resubmitted manuscript along with supporting letter from my study site.

I have mentioned all the genuine and true justification from my side, but the final decision will be yours.

Thank you

Regards

Dipsikha Aryal

Nepal
