# Supplementary material for: Predictors to Intensive Care Unit admission among patient with coronavirus disease in Sukraraj Tropical and Infectious Disease Hospital, Nepal: A case-control study
Source: PLOS Glob Public Health. 2024 Mar 21;4(3):e0002516. doi: 10.1371/journal.pgph.0002516 (PMC10957074; doi:10.1371/journal.pgph.0002516)
Supplement: Attachment — Submitted filename: Response to Reviewers.docx [file pgph.0002516.s004.docx]

Dear Editor and Reviewers,

Thank you so much for this wonderful consideration and we highly appreciate your meticulous review and constructive comments and suggestion. We have revised our manuscript incorporating your comments and suggestions. Here we write a rebuttal letter that responds to each point raised by the Editor and Reviewers. Please kindly consider our responses.

| **Comments** | **Response** |
| --- | --- |
| **Journal requirements** | |
| 1. In the ethics statement in the Methods, you have specified that verbal consent was obtained. Please provide additional details regarding how this consent was documented and witnessed, and state whether this was approved by the IRB. | The verbal consent was taken and documented by the following ways:  At first researcher collected list of participants meeting the inclusion criteria with contact details. We have directly contacted with each respondent, at first introduction of researcher and the main aim of the study were clearly explained, interview will last for around 10 – 15 minutes were clarified. Asked respondents about their most favorable time and contacted accordingly.  Participants information sheet was prepared during the time of proposal development, all the information to respondents about voluntary participation and confidentiality were provided in Nepali language. The way of taking consent were consistent to all the respondents. The date and time of consent provided by the respondents is recorded by researcher. This is approved by Institutional ethical review committee of BPKIHS (Reference number 236/078/079-IRC).  The main reason for verbal consent with the retrospective nature of study.  If required for further confirmation and assurance proposal submitted to IRC and participants information sheet can be uploaded. |
| 2. Please provide separate figure files in .tif or .eps format only and remove any figures embedded in your manuscript file. Please also ensure all files are under our size limit of 10MB. | Yes. We have incorporated as indicated. |
| 3. We have noticed that you have uploaded Supporting Information files, but you have not included a list of legends. Please add a full list of legends for your Supporting Information files after the references list. | Legends added for flow chart. |
| 4. Please amend your Data Availability Statement and indicate where the data may be found. | Data can be found in .osf, link of data osf attached below.  https://osf.io/az4wr/ |
| **Reviewer’s comment** | |
| **Reviewer # 1** | |
| 1. Methods section needs restructuring. Would benefit from a list of inclusions and exclusions early on, to include age restrictions and outcome (in-hospital death). Sample selection: Line 193 From my understanding, the 99 patients were not randomly selected. Rather, they identified ICU admissions with COVID-19 (who survived discharge) and then matched them to other patients with admitted to the ward with COVID-19. Lines 109-110: I think they are saying every 2nd or every 3rd. It is probably every 3rd patient was reviewed. | Inclusion and exclusion criteria have been added in line 147 – 167 of revised manuscript.  Line 193  Response: Only 33, that is ICU admitted patients were randomly selected according systematic random sampling. Rest 66 control were selected as per age and sex matched with case.  Line 109 – 110  Response: Yes, every 3^rd^ file was reviewed for ICU admitted case. Which is done via systematic random sampling method. |
| Lines 114-115. They mention “inclusion criteria”, but I do not see where those are listed. I think they are trying to say they selected 2 controls based on each case. Analysis: Lines 242-245 belong in the methods section ”variables that were significantly associated with ICU admission, with a p-value 243 less than or equal to 0.2 in bivariate analysis were taken for multivariate logistics regression 244 analysis. Variables with expected count less than 5 were not included in multivariate logistics 245 regression analysis.” | Line 114-115  Response: Thank you for the suggestion. Inclusion and exclusion criteria have been added in line 147 – 167 of revised manuscript.  Lines 242-245  Response: Reanalysis of data has performed; conditional logistics regression has done as per suggestion instead of binary logistics regression.  Independent variables found statistically significant (p<0.05) in bivariate analysis were only further utilized in conditional logistics regression.  The “p-value less than or equal to 0.2 in bivariate analysis were taken for multivariate logistics” this statement has been removed in revised manuscript line number 280 – 283. |
| 2. Lines 120-126 these are results (as per Figure 1). | Response: Yes, this is explanation of figure 1. |
| 3. Figure 1- it is surprising that all deaths occurred in ward patients. They should check that. | Response: As mentioned on line 133 – 134 of revised manuscript, the number 105 deaths are the total death during the course of treatment in hospital. The arrow in flow chart gives the unclear information and it shows that death occurred in ward only, which in fact is not true. The arrow is restructure as per comments.  In conclusion, 105 is total death in hospital during six months including both ICU admitted and general admission. |
| Also, why are the ICU patients separated into the ventilator group? Were those cases ineligible for the study as well? | Response: Patient who required ventilator were also included in the study. The ventilator group were separated in flow chart just to make clear picture about the ICU admitted group.  Flowchart is restructured and uploaded as .tif file. |
| 1. Recall bias – Data collection occurred in June – July 2022. The population contacted were in hospital between July 2021 and Jan 2022. This suggests telephone interviews asking about symptoms and preventative measures in the 2 weeks prior to their hospitalisation occurred between six months and a year after their hospitalisation. Preventative measures recall may be worse and/or biased for ICU patients compared to ward patients. Some variables are likely okay to collect six months after hospitalisation– e.g. smoking, comorbidities and vaccination status. I would not conclude anything, however, about other variables due to bias. | Response: The main limitation of the study with retrospective design is recall bias, in this study as well main limitation is recall bias. However, researcher has tried several techniques to minimize the recall bias, administration of structured questionnaire and interview to both case and control, consistency in the pattern of preventive practice questions, also the direct leading question was avoided.  Limitation of this study is mentioned in line 414 – 415 of revised manuscript. |
| 2. Data analysis: The analysis they did was flawed, hence I cannot yet believe the findings. Logistic regression model likely needed to be conditional due to the matching procedure. Inclusion of the matching variables (age and sex) is required at a minimum for matched studies. How was inter-dependence of variables assessed? Eg. Smoking and preventative practices? Smoking and sex? Vaccination status and smoking? With a sample of only 100 in total, no more than 5 or 6 covariates should be included. Sex and age need to be included, then they can try and build a parsimonious model with other variables with a large B-coefficient, after assessing for interdependence/ intercorrelations. Did they test COPD in multivariable model? And COPD was likely associated with smoking. Hence they need to include the interaction term of COPD*smoking or pick the better predictor of the two. The Nagelkerke R2 is very high. I would be surprised if the variables in the model explained that much of the variance. Furthermore, duration of hospital stay isn’t a predictor of ICU admission, it’s likely a result of ICU admission. That should not be included. | Response: Thank you for the comment. This comment has been addressed, according to suggestion. Re analysis of data set has performed, conditional logistic regression has done instead of binary logistics regression. Results are presented in table 6 of revised manuscript. |
| They discuss “severity at admission” in the methods section but do not present that data anywhere in the tables. It would be interesting to see that data, especially if the ward patients were the ones that were dying. Did patients with a “do not resuscitate” plan go to the ward directly? | Response: Thank you for the comment, sir/ma’am. The disease severity at the time of hospital admission is added in table 3 with diagnosis (recorded from the hospital file) and description. |
| Age categories would also be helpful to see, e.g. 18-39 years, 40-59 years, 60-79 years and 80+ years. | Response: Age group according to category 18-39 years, 40-59 years, 60-79 years and 80+ years is added in table 1. |
| **Reviewer #2** | |
| Minimum data was not provided, therefore it is impossible to verify the results. | Thank you for the comment. Data is made available in data availability system. |
| Study site and population not properly described. | Detail description of study site in line 98 - 107 and population in line 147 – 167 has been added in methodology section of revised manuscript. |
| The flowchart contains errors, as the numbers do not add up. The flowchart follows a cohort format, which is an inappropriate study design. It is recommended to approach the work as a retrospective cohort design or a cross-sectional analysis instead of a case-control study. | Total COVID-19 patients during six months = 713  Excluding <18 years (713 – 15 = 698)  Among 698 (ICU admitted = 222, General admission = 371) (Remaining 105 total death during the course of treatment)  That is 222 + 371 + 105 = 698  Among (222 + 371 = 593), initially selected 141 patients from 593, within them 33 case and 66 control were enrolled in study remaining were excluded due to unmatched.  Flowchart is restructured and submitted as .tif file. Patient enrollment criteria is clearly written in descriptive form and flowchart is drawn to make clear picture. If flowchart makes information confusing it can be deleted. |
| What was the power of the study calculated? The sample size (N) appears too small. | Taking reference of case control study done in Spain on 2021 (ref 3),  proportion of cases (p1) is 33.33% and proportion of control (p2) is 66.67%.  Formula, n= (Z _α/2_ + Z _β_) ^2^ (p_1_q_1_ + p_2_q_2_) / (p_1_-p_2_) ^2^  p_1_= 33.33% that is 0.33,  q_1_ = 0.67 p2= 66.67% that is 0.66,  q_2_= 0.34  Z _α/2_ = 1.96 at 95% Confidence Interval  Z _β_= 0.84 for 80% power  Now using formula,  n= (1.96 + 0.84)^2^ (0.33 * 0.67 + 0.66 * 0.34) / (0.33-0.66)^2^  = 7.84 (0.2211 + 0.2244) /0.1089  = 3.492 / 0.1089  = 32.07 ~ 33  Therefore, the calculated sample size for cases was 33 and control was 66 (1:2 ratio). Total number of samples was 99. |
| The study sample size was small; consequently, the statistical results yielded extremely wide confidence intervals. While these intervals allow us to determine whether a variable is associated with the model, they do not facilitate additional interpretations that contribute to understanding the risk probability. | Thank you for the comment. Yes, we did re-analysis of data set. Conditional logistic regression is performed instead of binary logistics, with the proper statistical technique the confidence interval is not wide.  This is the final results with confidence interval:  Patients with elevated Blood pressure (AOR = 2.22; 95% CI 1.05 – 4.71, p 2.2= 0.015), Abnormal CRP (AOR = 2.92; 95% CI 1.24 – 6.84, p = 0.012) at the time of hospital admission were more likely to get admitted to ICU. Patient with poor preventive practice (AOR = 3.34; 95% CI 1.19 – 9.31, p=0.02) more likely to get admitted to ICU than patient with good preventive practice.  This is incorporated in revised manuscript. |
| The abstract mentions "The adjusted multivariable logistics regression," but this information is not thoroughly explained in the methods section. Furthermore, the "multivariable logistics regression" only appears in Table 5, where it is referred to as 'binary logistic regression'. It is unclear whether the authors used binary or multiple logistic regression. | This comment has been addressed and incorporated in revised manuscript.  For bivariate analysis chi-square test and Mann- Whitney U test was performed.  Reanalysis of data has done and conditional logistic regression is performed in revised manuscript. |
| Report the number of instances of "missing data" for each variable. | Missing data were excluded prior to data analysis. |
| Include both crude and adjusted odds in the same Table 5. | We have incorporated COR and AOR in the same table, which is table number 6 in revised manuscript. |
| The reference levels for each variable in the logistic regression (Tables 3 & 4) are not clearly specified. | Thank you for the comment. Reference category has been added in each variable which is denoted by asterisk (*) symbol.  In the revised manuscript, table number 4 and 5. |
| For the variable "COVID-19 vaccination" (Yes/No), the reference level should be set as "Yes." This adjustment allows the "No" value to indicate the significantly increased risk for non-vaccinated individuals being hospitalized in the ICU. | Yes, the reference category is yes in “COVID-19 vaccination”.  Change is incorporated in revised manuscript. |
| Line 371: The sentence "result may be generalized only to patient under similar setting" is incorrect. The study's results cannot be generalized to patients under similar settings. The results of the study can only be generalized to patients within the same hospital. | Corrected as per suggestion. |
| **Minor Changes** | |
| Table 4 is incorrectly numbered. | Corrected. |
| In Table 5, provide the number of cases and controls for each variable. | Number of cases and control added, which is in table 6 of revised manuscript. |
| Line 51: Change "pandemic in March 2020(1)" to "pandemic in March 2020 (1)." | Changed incorporated in revised manuscript in line 52. |
| Line 55: Change "major symptoms like fever, cough, dyspnea" to "major symptoms such as fever, cough, and dyspnea." | Changed incorporated in revised manuscript in line 55. |
| Line 56: Change "cutaneous manifestations are common clinical features of this disease (2)" to "cutaneous manifestations are common clinical features of this disease (2)." | Same |
| Line 64: Change "The first case of coronavirus was seen in a Nepalese Citizen returning" to "The first case of coronavirus was observed in a Nepalese citizen returning." | Changed incorporated in revised manuscript in line 64. |
| Line 69: Change "endorsed in Nepal from 25th March 2020 (6)" to "endorsed nationwide in Nepal on March 25, 2020 (6)." | Changed incorporated in revised manuscript in line 69. |
| Line 242: Change "variables that were significantly associated with ICU admission" to "variables significantly associated with ICU admission." | Changed incorporated in revised manuscript in line 290. |
| Line 104: Change "public hospital with High Dependency Unit" to "public hospital with a High Dependency Unit." | Changed incorporated in revised manuscript in line 116. |
| Line 259: Change "COVID-19 infected patients was found to be 31.13%" to "The proportion of COVID-19 infected patients was found to be 31.13%. | Changed incorporated in revised manuscript in line 302 – 303. |

Thank you

Corresponding author
