# Supplementary material for: Predictors to Intensive Care Unit admission among patient with coronavirus disease in Sukraraj Tropical and Infectious Disease Hospital, Nepal: A case-control study
Source: PLOS Glob Public Health. 2024 Mar 21;4(3):e0002516. doi: 10.1371/journal.pgph.0002516 (PMC10957074; doi:10.1371/journal.pgph.0002516)
Supplement: Attachment — Submitted filename: 2nd Response to reviewer.docx [file pgph.0002516.s006.docx]

Dear Editor and Reviewers,

Thank you so much for this wonderful consideration and we highly appreciate your meticulous review and constructive comments and suggestion. We have revised our manuscript incorporating your comments and suggestions. Here we write a rebuttal letter that responds to each point raised by the Editor and Reviewers. Please kindly consider our responses.

| **Journal Requirement’s** | |
| --- | --- |
| **Comments** | **Response** |
| 1) In the ethics statement in the Methods, you have specified that verbal consent was obtained. Please provide additional details regarding how this consent was documented and witnessed, and state whether this was approved by the IRB. | The main reason for verbal consent with the retrospective nature of study.  The verbal consent was taken and documented by the following ways:  At first researcher collected list of participants meeting the inclusion criteria with contact details. We have directly contacted with each respondent, at first introduction of researcher and the main aim of the study were clearly explained, interview will last for around 10 – 15 minutes were clarified. Asked respondents about their most favorable time and contacted accordingly.  Participants information sheet was prepared during the time of proposal development, all the information to respondents about voluntary participation and confidentiality were provided in Nepali language. The way of taking consent were consistent to all the respondents. The date and time of consent provided by the respondents is recorded by researcher. This is approved by Institutional ethical review committee of BPKIHS (Reference number 236/078/079-IRC).  If required for further confirmation and assurance proposal submitted to IRC and participants information sheet can be uploaded.  Consent was witnessed by Mr Khilnath Gautam, who was medical recorder at Sukraraj Tropical and Infectious Disease Hospital during the time of data collection. Hospital file was taken, reviewed and participants were contacted in front of him. If required contact number and email of medical recorder can be made available. |
| 2) Please review your reference list to ensure that it is complete and correct. If you have cited papers that have been retracted, please include the rationale for doing so in the manuscript text, or remove these references and replace them with relevant current references. Any changes to the reference list should be mentioned in the rebuttal letter that accompanies your revised manuscript. If you need to cite a retracted article, indicate the article’s retracted status in the References list and also include a citation and full reference for the retraction notice. | Reference list is completed and correct. Retracted papers are not cited. |
| **Reviewer’s comment** | |
| **Reviewer # 1** | |
| 1) The study’s sample size calculation doesn’t make sense. The “disease” is ICU admission in this study, the “exposure” is any of: smoking, high bp, etc. To do the sample size you would pick one of these (whichever is the most important in your opinion) and have an estimate of how common that exposure one in the cases and the controls. Then you could get a valid sample size. Since the study didn’t do sample size calculation in this manner, I recommend deleting the sample size calculation paragraphs. | Thank you for the suggestion. Sample size calculation paragraph has been deleted as per recommendation in 2^nd^ revised manuscript. |
| 2) The period of review was 16th July 2021- 14th January 2022 according to page 6 line 108. | Yes, the period of review was from 16^th^ July 2021 – 14^th^ January, 2022 which is in page 7 line 123 of second revised manuscript. |
| 3) The authors excluded patients who died. Please explain why this is justified? If it is because you can’t ascertain the “preventative measures”, again, this is a flaw in the study design. Any findings of the study can be interpreted as valid only for people who survive COVID-19. | Yes, of course preventive measures can’t be ascertained. The main reason was for accuracy, reliability of study and to avoid bias. |
| 4) I appreciate the authors use of conditional logistic regression modelling for matched data. However, the authors also have to include the matching variables – age and sex- in the model for the model to be accurate. The results for age and sex don’t matter, but they may effect the other exposures of interest and need to be included in the model. |   This screenshot is the output from SPSS after including matching variables in model. The results from both model shows same variables to be found statistically significant. |
| 5) In results, the authors state that 99 patients were randomly selected from the population of PCR+ patients. This is not the case. 33 patients admitted to ICU who were able to be contacted post discharge were selected randomly from all patients admitted to ICU and able to be contacted post-discharge, then these were matched 2:1 to other patients admitted to the general ward who were able to be contacted post discharge. | Thank you for suggestions. That line from results has been rewritten in line 228 of 2^nd^ revised manuscript.  This has been rewritten as; A total of 33 ICU admitted patients and 66 patients admitted in general ward were selected from 713 patients who were hospitalized during the study period in participating centers. |
| 6) Tables 2 to 5 present crude odds ratios. This should be qualified as such in the titles. Also, whether or not it is valid to present crude odds ratios for a matched study is debatable. In tables 2-5, the authors should consider presenting a) crude odds ratios and b)AOR for the variable, adjusting for age and sex. | Crude odds ratio has been calculated from Bivariate analysis. AOR has been calculated in only those variables which were significant in bivariate analysis.  COR and AOR has been incorporated and presented in same table of 2nd revised manuscript. |
| **Reviewer #2** | |
| 1) English requires revision. Several phrases lack proper punctuation, making it difficult to read. I have pointed out some adjustments, but only a few. The English is well-written and mostly easy to read, but an extensive grammar review is required. | Thank you for the suggestions. Grammar review has been done and corrected as per suggestions. |
| 2) The flowchart still needs adjustments (check comments in the PDF-reviewed file). I wonder if it is necessary to be in the text. | Exclusion due to death has been added in flowchart. Out of total admitted patients initially reviewed 141 files were reviewed, among them 33 cases matched with 66 controls. The calculated sample size was reached due to which 42 initially selected group excluded.  If the flowchart is creating confusion among readers, it can be removed as per suggestions. |
| 3) Tables need adjusting. Tables 2 to 5 can be merged. | Table 2 and 4 has been merged and uploaded as table 2 in 2^nd^ revised manuscript. Additionally, COR of table 5 has been incorporated in table 6. So, table 5 and 6 has been renamed as table 4 in 2^nd^ revised manuscript. |
| 4) Check manuscript formatting at [link1] and [link2]. | Done and corrected as per suggestions. |
| 5) In the bivariate results, it is not clear why only blood pressure, C-reactive protein, and COVID-19 vaccination variables from Table 4 were included in the conditional logistic regression? | To avoid the problem of multicollinearity and for reliable findings, variables with expected count greater than 5 were only included in conditional logistic regression. |
| 6) Standardize the use of 'full-written-words' and abbreviations. | Necessary correction has been performed as suggestion. |
| 7) Add an additional file with maps/geographical visualization of the country/state/province and hospital location. That will help foreign readers better understand the study's epidemiological aspects. I suggest using the following model: [link3]. | Map added as supporting documents, Sukraraj Tropical and Infectious Disease hospital is marked with red colour. |
| 8) Table 6 is not in a good format; I suggest using the following model: [link4]. | Table 5 and 6 has been merged as presented with COR and AOR with p value. |
| 9) There is room for improvement in the last paragraph of the discussions section on the matter of 'study generalization,' and in the conclusions section, provide more direct conclusions on how to use the results found to improve public health actions/strategies/policies, although it is already implicit in discussions | Incorporated as per suggestions. |

Thank you

Corresponding author
