# Supplementary material for: Predictors to Intensive Care Unit admission among patient with coronavirus disease in Sukraraj Tropical and Infectious Disease Hospital, Nepal: A case-control study
Source: PLOS Glob Public Health. 2024 Mar 21;4(3):e0002516. doi: 10.1371/journal.pgph.0002516 (PMC10957074; doi:10.1371/journal.pgph.0002516)
Supplement: Attachment — Submitted filename: 3rd response to reviewer.docx [file pgph.0002516.s008.docx]

Dear Editor and Reviewers,

Thank you so much for this wonderful consideration and we highly appreciate your meticulous review and constructive comments and suggestion. We have revised our manuscript incorporating your comments and suggestions. Here we write a rebuttal letter that responds to each point raised by the Editor and Reviewers. Please kindly consider our responses.

| **Journal’s requirements** | |
| --- | --- |
| **Comments** | **Response** |
| 1. In the ethics statement in the Methods, you have specified that verbal consent was obtained. Please provide additional details regarding how this consent was documented and witnessed, and state whether this was approved by the IRB" | The main reason for verbal consent with the retrospective nature of study.  The verbal consent was taken and documented by the following ways:  At first researcher collected list of participants meeting the inclusion criteria with contact details. We have directly contacted with each respondent, at first introduction of researcher and the main aim of the study were clearly explained, interview will last for around 10 – 15 minutes were clarified. Asked respondents about their most favorable time and contacted accordingly.  Participants information sheet was prepared during the time of proposal development, all the information to respondents about voluntary participation and confidentiality were provided in Nepali language. The way of taking consent were consistent to all the respondents. The date and time of consent provided by the respondents is recorded by researcher. This is approved by Institutional ethical review committee of BPKIHS (Reference number 236/078/079-IRC).  If required for further confirmation and assurance proposal submitted to IRC and participants information sheet can be uploaded.  Consent was witnessed by Mr Khilnath Gautam, who was medical recorder at Sukraraj Tropical and Infectious Disease Hospital during the time of data collection. Hospital file was taken, reviewed and participants were contacted in front of him. If required contact number and email of medical recorder can be made available. |
| **Reviewer’s comment** | |
| **Reviewer # 2** | |
| I am satisfied with the corrections made. However, after reading the comments from reviewer 1, I am uncertain whether the description of the sampling calculation should indeed be included in the main text. The flowchart still requires improvements as it lacks information, and the mathematics do not align. Please check the additional reviewer file to see the infor that are missing. The repositioning of the 4th box should be under box 3. Additionally, the flowchart is not visually pleasing, and the arrows are not aligned. Kindly adjust the flowchart again or consider removing it. | Thank you for the comments. If flowchart is creating confusion among readers, sampling techniques in flowchart can be removed with its description included in main text.  Flowchart has been modified into this structure as per suggestion of previous reviewers.  **Mathematics**  Total COVID-19 patients during six months = 713  Excluding <18 years (713 – 15 = 698)  Among 698 (ICU admitted = 222, General admission = 371) (Remaining 105 total death during the course of treatment)  That is 222 + 371 + 105 = 698  Among (222 + 371 = 593), initially selected 141 patients from 593, within them 33 case and 66 control were enrolled in study remaining were excluded due to unmatched. |
| The map figure lacks subtitles. What do the red GPS markers represent? And what about the blue ones? What do the blue-painted zones indicate? The map also lacks a scale and a compass rose. I suggest inserting a small figure of the country, indicating where the study is conducted. Please refer to the example in the file I sent. | GPS map has been added in second revised version of manuscript as per the suggestion of reviewers.  The exact map of hospital with mentioned details can’t be found. I think map can also be removed as per your suggestions. |
| **Reviewer #3** | |
| 1. The main issue this article is dealing with is widely studied and a significant number of articles about it are published so far. The fact that in Nepal this might be the first study of its kind but this is not relevant enough for publishing. | Thank you for the comments. This will be the first case control study in Nepal showing comparative results between ICU admitted and non-admitted.  Yes, there are significant number of publications with the main outcome COVID-19 related death, but there are only few articles of COVID-19 with the outcome ICU admission and general admission. This study have excluded deaths and find predictors among survivors only. |
| 2. The Introduction section should inform the reader about the knowledge that we already have about the research subject, and after that to explain what were the reasons that this study was conducted. | Thank you for the comments. Introduction section has been reorganized in the suggested pattern, the main reason for this study is written from line 120 – 127 in revised manuscript. |
| The Patients and Methods section has to much better organized and written. Authors mention inclusion criteria, and those criteria are not explained; what was the reason to exclude 105 COVID-19 patients that died in non-ICU wards?; authors mention the stratification of patients according to the disease severity, yet it is not present in the text later; how can the length of stay in the hospital be used as a predictor for the ICU admission? | Methods section has been organized and re-written as per recommendations.  The main outcome variable of study was to compare predictors between making ICU admission (cases) and General ward admission (control). All the patients who died during hospital treatment was excluded from study.  This study was conducted only among patient those who survived. The reason for exclusion was death of 105 patients during the course of treatment. Details is below:  **Mathematics**  Total COVID-19 patients during six months = 713  Excluding <18 years (713 – 15 = 698)  Among 698 (ICU admitted = 222, General admission = 371) (Remaining 105 total death during the course of treatment)  That is 222 + 371 + 105 = 698  Among (222 + 371 = 593), initially selected 141 patients from 593, within them 33 case and 66 control were enrolled in study remaining were excluded due to unmatched.  Disease severity has been categorized as ICU admission and general ward admission.  The variable length of stay was not analyzed further in statistical analysis. |
| The methodology for the questionnaires use has to be properly explained; in this study high blood pressure at the admission was a predictor for the ICU admission, yet no comorbidities, including hypertension was not a predictor for the ICU admission...? | Details about questionnaire has been described from line 315 – 324. The use of this questionnaire was to assess the application of preventive practice before COVID-19, also the questionnaire was designed in similar setting like Nepal.  Regarding comorbidities the detail explanation is described from line **482 – 513** of revised manuscript. |
| The Discussion section has to be better organized and much better written, avoiding speculations like the one when authors try to explain the differences in natural immunity between people of different countries. | Thank you for suggestion. Corrected as per suggestion. |
| The Conclusions section should be re-written after the manuscript revision. | Thank you for suggestion. Corrected and re-written as per suggestion. |
| 3. The report is written in rather poor English language and it is highly recommended that the manuscript should be revised by a native English language speaker. | The whole manuscript has rewritten and submitted after intensive grammar and language review. Thank you |
